# Genome-wide characteristics of *de novo* mutations in autism

**DOI:** 10.1038/npjgenmed.2016.27

**Published:** 2016-08-03

**Authors:** Ryan KC Yuen, Daniele Merico, Hongzhi Cao, Giovanna Pellecchia, Babak Alipanahi, Bhooma Thiruvahindrapuram, Xin Tong, Yuhui Sun, Dandan Cao, Tao Zhang, Xueli Wu, Xin Jin, Ze Zhou, Xiaomin Liu, Thomas Nalpathamkalam, Susan Walker, Jennifer L Howe, Zhuozhi Wang, Jeffrey R MacDonald, Ada JS Chan, Lia D’Abate, Eric Deneault, Michelle T Siu, Kristiina Tammimies, Mohammed Uddin, Mehdi Zarrei, Mingbang Wang, Yingrui Li, Jun Wang, Jian Wang, Huanming Yang, Matt Bookman, Jonathan Bingham, Samuel S Gross, Dion Loy, Mathew Pletcher, Christian R Marshall, Evdokia Anagnostou, Lonnie Zwaigenbaum, Rosanna Weksberg, Bridget A Fernandez, Wendy Roberts, Peter Szatmari, David Glazer, Brendan J Frey, Robert H Ring, Xun Xu, Stephen W Scherer

**Affiliations:** 1 The Centre for Applied Genomics, Genetics and Genome Biology, The Hospital for Sick Children, Toronto, ON, Canada; 2 Deep Genomics Inc., Toronto, ON, Canada; 3 BGI-Shenzhen, Yantian, Shenzhen, China; 4 Department of Electrical and Computer Engineering, University of Toronto, Toronto, ON, Canada; 5 Department of Molecular Genetics, University of Toronto, Toronto, ON, Canada; 6 Program in Genetics and Genome Biology, The Hospital for Sick Children, Toronto, ON, Canada; 7 Center of Neurodevelopmental Disorders (KIND), Pediatric Neuropsychiatry Unit, Karolinska Institutet, Stockholm, Sweden; 8 Google Genomics, Google Cloud Platform, Google Inc., Mountain View, CA, USA; 9 Verily Life Sciences, South San Francisco, CA, USA; 10 Autism Speaks, Princeton, NJ, USA; 11 Department of Molecular Genetics, Paediatric Laboratory Medicine, The Hospital for Sick Children, Toronto, ON, Canada; 12 Bloorview Research Institute, University of Toronto, Toronto, ON, Canada; 13 Department of Pediatrics, University of Alberta, Edmonton, AB, Canada; 14 Department of Paediatrics, University of Toronto, Toronto, ON, Canada; 15 Disciplines of Genetics and Medicine, Memorial University of Newfoundland, St John’s, Newfoundland, NL, Canada; 16 Provincial Medical Genetic Program, Eastern Health, St John’s, Newfoundland, NL, Canada; 17 Autism Research Unit, The Hospital for Sick Children, Toronto, ON, Canada; 18 Child Youth and Family Services, Centre for Addiction and Mental Health, Toronto, ON, Canada; 19 Department of Psychiatry, University of Toronto, Toronto, ON, Canada; 20 Donnelly Centre for Cellular and Biomolecular Research, University of Toronto, Toronto, ON, Canada; 21 McLaughlin Centre, University of Toronto, Toronto, ON, Canada

## Abstract

*De novo* mutations (DNMs) are important in autism spectrum disorder (ASD), but so far analyses have mainly been on the ~1.5% of the genome encoding genes. Here, we performed whole-genome sequencing (WGS) of 200 ASD parent–child trios and characterised germline and somatic DNMs. We confirmed that the majority of germline DNMs (75.6%) originated from the father, and these increased significantly with paternal age only (*P*=4.2×10^−10^). However, when clustered DNMs (those within 20 kb) were found in ASD, not only did they mostly originate from the mother (*P*=7.7×10^−13^), but they could also be found adjacent to *de novo* copy number variations where the mutation rate was significantly elevated (*P*=2.4×10^−24^). By comparing with DNMs detected in controls, we found a significant enrichment of predicted damaging DNMs in ASD cases (*P*=8.0×10^−9^; odds ratio=1.84), of which 15.6% (*P*=4.3×10^−3^) and 22.5% (*P*=7.0×10^−5^) were non-coding or genic non-coding, respectively. The non-coding elements most enriched for DNM were untranslated regions of genes, regulatory sequences involved in exon-skipping and DNase I hypersensitive regions. Using microarrays and a novel outlier detection test, we also found aberrant methylation profiles in 2/185 (1.1%) of ASD cases. These same individuals carried independently identified DNMs in the ASD-risk and epigenetic genes *DNMT3A* and *ADNP.* Our data begins to characterize different genome-wide DNMs, and highlight the contribution of non-coding variants, to the aetiology of ASD.

## Introduction

Autism spectrum disorder (ASD), a neurobehavioral condition characterised by atypical development of social-communication, and the presence of restrictive interests and repetitive behaviours, can have a genetic basis.^1^ ASD exhibits extensive clinical and genetic heterogeneity with high heritability^2^ and recurrence risk,^3^ and males are affected more often than girls (~4:1).^4^ Copy number variations (CNVs),^5,6^ insertion–deletions (indels),^7,8^ and single nucleotide mutations^6,9^ have implicated >100 ASD susceptibility genes^5,6,10^ of variable penetrance and expressivity, some of which are making their way into clinical genetic testing,^11,12^ but most of which are still to be defined.^10^ Functionally, ASD-risk genes often converge in pathways that modulate synaptic transmission, chromatin remodelling and transcriptional regulation.^5,9^ Common genetic variants may also contribute to ASD.^13^

With over a decade of experience in genomic studies of ASD, the approach of searching for *de novo* mutations (DNMs) continually emerges as an effective method to initially sort through increasingly complex data sets.^14–17^ Due to previous technology limitations in resolution and cost, the vast majority of studies have interrogated the small (~1.5%) gene-coding segments of the genome. In a recent study, penetrant DNMs in genes were estimated to contribute to ASD in ~11% of parent–child trios (simplex) families.^6^ Even our own research using whole-genome sequencing (WGS)^7,18^ focused only on annotating genes, since sample sizes were insufficient to discern statistically relevant data from the larger non-coding regions (~98.5% of the genome).

Here, we developed new approaches to characterize DNMs from WGS data, with an emphasis on determining their origin and functional impact on non-coding DNA in ASD. Our most compelling data found that clustered DNMs in ASD mostly originated from the mother, and are often found adjacent to *de novo* CNVs. In addition, we found that coding and non-coding *de novo* point mutations in ASD are enriched in genes that are responsible for synaptic, translational and chromatin remodelling function. We have also demonstrated that these DNMs may have deleterious effects on the epigenetic profiles of individuals with ASD. Somatic mutations potentially relevant to ASD were also detectable in the WGS data.

## Results

### Detection of genome-wide DNMs

We performed WGS in 200 unrelated idiopathic ASD trio families (600 individuals) using the Illumina Hiseq 2000 technology (Illumina, San Diego, CA, USA). The families were selected based on the fact that the index case (proband) was the only individual in the family affected with ASD. Subjects met criteria for ASD based on the Autism Diagnostic Interview-Revised (ADI-R), the Autism Diagnostic Observation Schedule-Generic (ADOS) plus clinical evaluation. All probands were genotyped for CNVs using high-resolution microarrays (Supplementary Information).

Of the 200 probands, genomic DNA was obtained for 192, 4 and 4 subjects from whole-blood, lymphocyte cell line (LCL) and leucocytes, respectively. The average coverage relative to the hg19 reference sequence (non-N bases) was 99.7% or 32× (Supplementary Table 1). Using an improved DNM detection approach,^7^ we identified 9,774 germline DNMs. This represents 50.9 *de novo* single nucleotide variants (SNVs), 3.9 *de novo* indels and 0.052 *de novo* CNVs (defined as unbalanced changes >10 kb) per genome, and their validation rates were 95.7% (377 of 396), 100% (21 of 21) and 62.5% (10 of 16), respectively (Supplementary Figure 1; Supplementary Tables 2 and 3). In the exonic regions, there were 0.99 *de novo* SNVs, 0.1 *de novo* indels and 0.03 *de novo* CNVs (Supplementary Tables 2 and 3) per individual. We found an unusually high number of DNMs in four of the LCL samples (Supplementary Table 2), consistent with previous observations.^18,19^ We also found a shift of the allelic fraction (alternate reads over total reads) supporting the variant towards the lower end (Supplementary Figure 2), confirming that most of the DNMs were cell-line-derived mutations of a mosaic nature.^18^ These eight samples (including the four from leucocytes) were therefore removed from our analysis.

### Origin of DNMs

We performed phasing to determine the chromosome of origin of the DNMs (Supplementary Information) and determined that 75.6% of the *de novo* SNVs and 68.6% of the *de novo* indels originated from the father (Figure 2a; Supplementary Table 4). Consistent with previous reports,^7,20^ the number of germline DNMs was found to increase with paternal age (Pearson correlation test, *r*=0.4; *P*=4.2×10^−10^; Figure 2b), which is mostly attributed to the higher number of replication events in the older paternal gamete.^20^ However, we found no correlation between the number of *de novo* SNVs on the maternal allele and the maternal age, suggesting few DNMs were accumulated throughout life in female. The number of phased *de novo* indels was insufficient for robust statistical analysis, but we could demonstrate the total aggregate number of *de novo* indels was more significantly correlated with paternal rather than maternal age (Poisson regression *β* coefficient based on Student’s *t*-distribution, *P*=6.4×10^−3^ for paternal age and *P*=0.74 for maternal age; Supplementary Figure 3 and Supplementary Table 5).

We also found a substantial portion of DNMs clustered (⩾2 mutations occurring within a 20 kb segment) in the same individual (239 DNMs in Supplementary Table 6) (Figure 2c). This phenomenon has been described previously in Dutch population controls,^21^ and similarly we found that clustered DNMs have different sequence signatures than non-clustered ones (Supplementary Figure 4).^21^ Remarkably, 43.9% of them (105 out of 239 DNMs) clustered within 200 bp (Supplementary Table 6). One such cluster of DNMs was found in a known ASD-risk gene, *SYNGAP1;*^5,6,9^ two *de novo* events were identified in the coding region of the gene in ASD case 3-0438-000. These mutations result in a 12 bp to 7 bp substitution that removes a core splice site of the exon (Supplementary Figure 5).

Contrary to what was observed in the Dutch population controls where fathers contribute a majority of clustered DNMs (Supplementary Figure 6), in our ASD families, we found that the majority of the clustered DNMs originated on the maternal lineage (Fisher’s exact test, *P*=7.7×10^−13^; Figure 2a). We also validated this finding on re-analysis of our previously reported ASD WGS data (Supplementary Figure 6).^18^ In search of an explanation, we found that mutation rates have been reported to be increased near CNVs.^22^ Indeed, we found that the DNMs near the 10 *de novo* CNVs (±100 kb) found in our sample are significantly higher than the expected genome background (Binominal test, *P*=2.4×10^−24^; Figure 2d). This involved 11 DNMs (7 of the 11 DNMs were clustered DNMs described above) in 5 *de novo* CNVs, and they were all separated over 1 kb (Supplementary Table 7). Interestingly, there is a significant reduction of maternal contribution in DNMs separated >200 bp (68% maternal) than those separated <200 bp (88% maternal) (Fisher’s exact test, *P*=0.01). No significant difference was found for the origin of *de novo* CNVs (or rare-inherited) from the parents^5^ (Supplementary Table 7), so it is unlikely that maternal enrichment of clustered DNMs can be explained due to a higher *de novo* rate of CNV from the mother. Instead, it may be caused by sex-based differences on DNA repair mechanisms during gametogenesis^23^ (Supplementary Table 7). Also, the fact that not all of the clustered *de novo* point mutations were found in *de novo* CNVs may be partially due to the false negative rate of CNV detection from current WGS technology.^18,24^

### Somatic mutations

**Figure 1:**
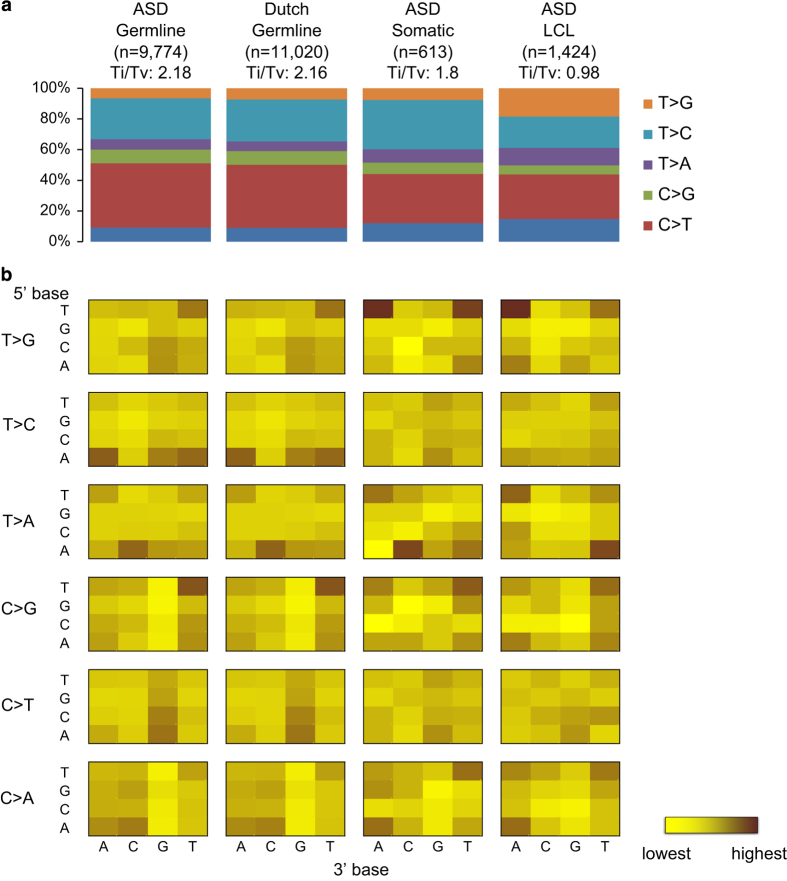
Sequence context of regions with *de novo* mutations. (**a**) Transition (Ti) to transversion (Tv) ratio of different kinds of *de novo* mutations found in: germline of ASD, germline of Dutch population controls, somatic events of ASD and lymphocyte-derived cell line (LCL) of ASD. (**b**) Sequence context of the base substitution mutation spectra for different *de novo* mutations. Each of the 96 mutated trinucleotides (mutated position at centre) from each cohort is represented in a heatmap (intensity of colour correspond to frequency of each mutation). The 5′ base to the mutated site is shown on the vertical axis, while the 3′ base is shown on the horizontal axis.

**Figure 2:**
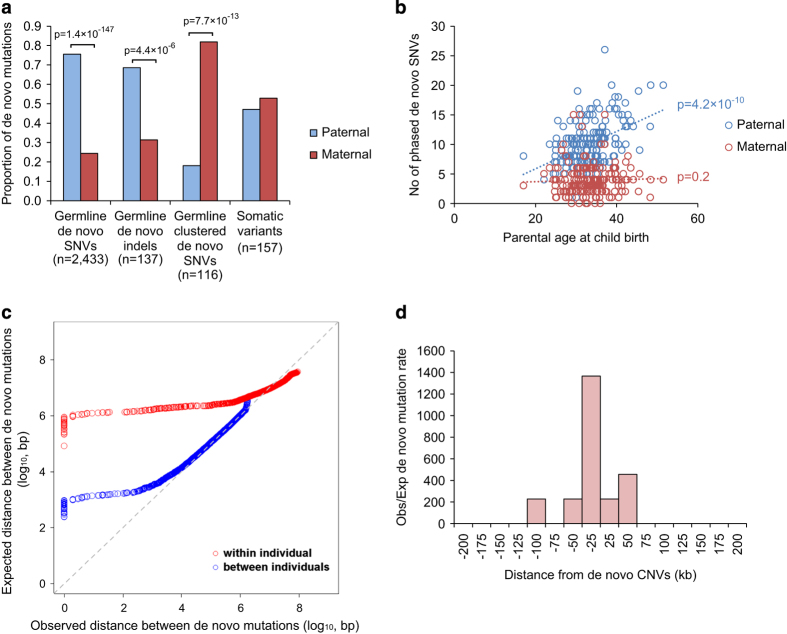
Origins of *de novo* mutations in ASD. (**a**) Parent-of-origin of germline and somatic variants. Number of germline *de novo* SNVs and *de novo* indels derived from the father was significantly higher than that from the mother. On the other hand, there are significantly more clustered (within 20 kb) germline *de novo* mutations originating from the mother than from the father, while somatic mutations can be found in similar proportion from both parents. (**b**) Number of *de novo* SNVs found on the paternal allele increases with the age of father, but there is no correlation between the number of *de novo* SNVs found on the maternal allele and the age of the mother. (**c**) Distance between *de novo* mutations is shorter than expected for a subset of *de novo* mutations both between and within individuals. (**d**) Mutation rate is significantly higher than the background within 100 kb flanking the *de novo* CNVs.

Among the DNMs, we found that there is a substantial portion of variants with a lower allelic fraction (<33%, 2 s.d. from the mean). We compared the sequence context of the DNMs with <33% allelic fraction to the rest of the variants (Supplementary Figure 7). We found that their sequence context is similar to that of the LCL-derived variants (Figure 1), suggesting that they may be generated by a similar mechanism. Therefore, most of these DNMs are likely to be somatic in origin, which is supported by the fact they were found almost equally from both maternal and paternal alleles (Figure 2a), and differ from what is seen in the constitutional genome. These correspond to 3.19 somatic mutations per genome and 0.036 per exome (Supplementary Table 2). One of these somatic mutations affects the *NRXN1*, a known ASD known ASD-risk gene^17,25^ (Supplementary Figure 8; Supplementary Table 3). Although the status of these mutations in the brain of carrier individuals would not be known, the relatively high allelic representation (16%) suggest they arose early in post-zygotic development and therefore may be extensively represented in cells throughout the body and therefore have phenotypic consequence.

### Functional characteristics of DNMs

To assess the potential functional effect of the DNMs identified in the ASD cohort, we compared them with the DNMs detected in a Dutch control population, in which the genomes of 258 parent–child trios (250 families) were sequenced with the same platform.^21,26^ These samples were collected without ascertaining on the basis of disease.^26^ We compared only the autosomal *de novo* germline SNVs from the ASD data because they were the only DNMs that were reported from that control population. While there is a difference in the sequence depth between our cohort (32×) and the control cohort (13.3×), we found that the sequence context associated with the DNMs was similar between the two (Pearson correlation test, *P*=5.8×10^−61^; Figure 1), which is not observed when using different sequencing platforms or DNM detection methods (Supplementary Figure 9). This observation suggests that there is no significant sequencing or detection bias between the cases and controls in this study. The high-validation rate of our DNM detected (95.7%) is also comparable to that of the controls (94.6% specificity). The difference in sequence coverage, however, can lead to variant detectability in regions with extreme GC content. Indeed, we found a minor difference in GC content in regions spanning DNMs between our cases and controls (Supplementary Figure 10). Therefore, we used a logistic regression test with GC content as a covariate to correct for this potential confounding effect (see Materials and methods).

**Figure 3:**
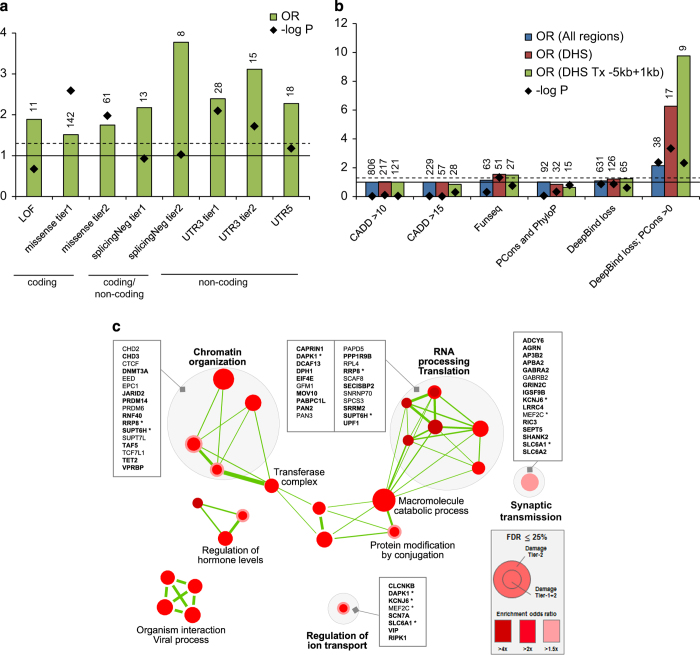
Functional impact of genome-wide damaging *de novo* mutations. (**a**) Damaging *de novo* mutations are significantly enriched in both coding (missense) and non-coding regions (splicingNeg, UTR3 and UTR5) in ASD compared with population controls. Definition of damaging tiers can be found in the Materials and methods. LOF, loss-of-function mutations; missense, missense mutations; splicingNeg, exon-skipping mutations predicted by SPANR; UTR, untranslated regions. Number of variants is indicated above each bar. Solid horizontal line indicates OR=1, and dash horizontal line represents *P*=0.05. (**b**) Non-coding *de novo* mutations in non-genic regions are significantly enriched in DNase I hypersensitive regions (DHS). Damaging *de novo* mutations predicted by ‘Deepbind loss; PCons>0’ are significantly enriched in ASD in general (All regions), but further enriched in DNase I hypersensitive regions and regions proximal to genes. DHS, DNase I hypersensitive sites; PCons, PhastCons; Tx, transcript. Number of variants is indicated above each bar. Solid horizontal line indicates OR=1, and dash horizontal line represents *P*=0.05. (**c**) Damaging *de novo* mutations are significantly enriched (false discovery rate ⩽0.25) in Gene Ontology defined pathways that are related to chromatin organisation, RNA processing and translation, synaptic transmission and others. Genes involved in the pathways are listed. Genes with DNMs in coding region are bolded. Asterisk represents gene that is found in more than one gene pathway.

Comparing the 9,774 germline DNMs from our ASD cohort (192 trios) with 11,020 DNMs from the Dutch control cohort at different genomic regions, we found that the DNM rate is higher at the 5′ untranslated region (5′-UTR) and the coding exons in ASD (Figure 3a). We further examined the *in silico* predicted effects of the DNMs. While loss-of-function (LOF) DNMs have a higher odds ratio compared with the control sample, they are not significantly enriched because of the small number of LOF mutations involved (Figure 3; Supplementary Table 8). On the other hand, we found a significant enrichment of *de novo* missense mutations in the ASD sample compared with controls (Figure 3a). This was not previously observed in the simplex proband–sibling comparison.^27^ Perhaps some of the supposedly unaffected siblings in families with ASD children were in fact at risk of ASD or other developmental phenotypes,^15^ a phenomenon that we have found previously.^7,18^

Beyond the coding region, we found that, in addition to the 5′-UTR, the 3′-UTR was significantly enriched with DNMs when we restricted our analysis to the conserved regions (Figure 3a). Although there is some enrichment of DNMs at the conserved long non-coding RNA, the difference did not reach the statistical significance (Supplementary Table 8). We have also applied a variant effect prediction tool we developed, SPANR,^28^ to annotate the effect of the variants at predicted splice sites (both exonic and intronic regions). We also found that the variants predicted with an exon-skipping effect (splicingNeg)^28^ represent the highest significant enrichment of variants in ASD compared with the controls (Figure 3a), while no significant enrichment was found in predicted benign missense (OR=1.2; *P*=0.27), synonymous (OR=1.19; *P*=0.65) and intronic (excluding predicted damaging) (OR=1; *P*=0.98) DNMs (Supplementary Table 8). For variants in the non-genic regions, we applied four different prediction tools and examined the burden of these ASD variants in different chromatin states from ENCODE^29^ and Epigenomic Roadmap^30^ (see Materials and methods and Supplementary Table 9). We found that DNMs were significantly predicted to lead to loss of transcriptional binding factors (Figure 3b). They were enriched in DNase I hypersensitive regions and proximal to genes. For example, a loss of KDM5B binding was found at the promoter of a candidate autism-risk gene, *EFR3A* (Supplementary Figure 11). Comparing 71 different human primary cell types or tissues, the effect of transcriptional binding factor loss was enriched in quiescent states of different brain regions (Supplementary Figure 12). Selecting a set of brain-specific enhancers without applying prediction algorithms, we also found a trend of enrichment in cases (OR=1.7, *P*=0.07). Taking together, putative non-coding DNMs that were significantly enriched in ASD represent 38% (93 out of 244) of the variants considered to be damaging (Table 1; Supplementary Table 10).

To evaluate the functional relevance of the predicted damaging DNMs, we compared the mutation burden between the ASD cases and the controls in the gene sets previously shown to be involved in ASD.^5,18^ Since it is still challenging to elucidate the target genes for linked non-coding variants in the non-genic regions, we focused our analyses on the DNMs found in gene-encompassing regions (exonic and intronic regions; 206 DNMs in total). Consistent with previous findings,^5,9^ we found that the predicted damaging DNMs in ASD samples have a significantly higher mutation burden in genes that are expressed in the brain, are FMRP targets, and other genes that are known to be involved in neurodevelopmental or behavioural phenotypes (Supplementary Figure 13).

To identify novel gene pathways that were enriched in the genes disrupted by the DNMs, we tested the mutation burden in all the gene sets listed in the Gene Ontology. We found a significant enrichment of variants in pathways involved in ‘chromatin organisation’, ‘RNA processing translation’ and ‘synaptic transmission’ among others (Figure 3c), which is largely consistent with the previous findings.^5,9^ These included many genes that are known to be involved in ASD, for example, *SHANK2*, *EIF4E* and *DAPK1*, further supporting the critical role of these pathways in ASD.

Importantly, we applied our previously developed tools to identify damaging non-coding variants. We showed that these predicted damaging non-coding variants were enriched in the splicing, 5′- and 3′-UTR, and together contribute 22.5% of the potential damaging DNMs examined (Table 1). Indeed, from the gene sets that were enriched with the pathways mentioned above, 29% (16 out of 56) of the genic variants involved were non-coding (Figure 3c), supporting the hypothesis in ASD that damaging non-coding variants may affect gene function in a manner similar to coding variants. We estimated that the damaging DNMs in genic regions (including coding and non-coding variants) contribute ~45% of the ASD cases in simplex families, which is largely consistent with that previously estimated.^27^

### Mutations altering epigenetic profiles

Given that DNMs in ASD can affect chromatin organisation, we performed DNA methylation profiling using Illumina Infinium array (Illumina, San Diego, CA, USA) of 185 probands for which whole-blood DNA was available to assess for epigenetic aberrations that might be mapped to the genomic sequence. Since mutations in ASD are highly heterogeneous, we speculated that samples having extreme epigenetic aberration would be rare. Therefore, we sought to identify samples with ‘rare methylation signatures’^31^ by detecting outliers from the overall DNA methylation pattern. To capture this effect, we developed a new approach called Methylation Outlier Sample Test (MOST; see Materials and methods).

**Figure 4:**
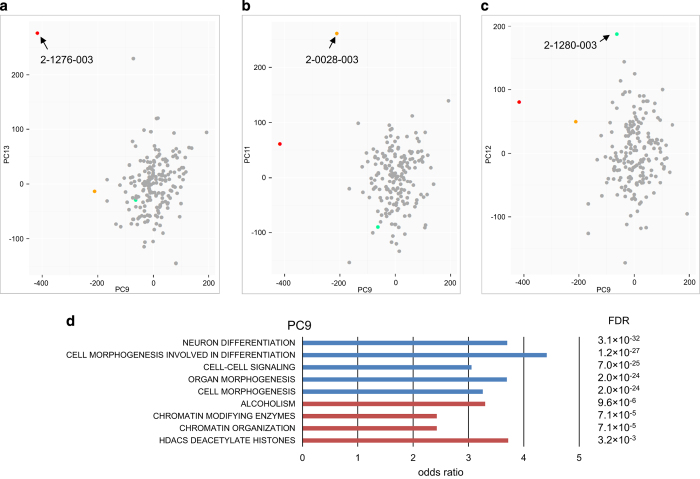
Sample outliers identified by the Methylation Outlier Sample Test (MOST). (**a**) Sample 2-1276-003, carrying a *de novo* damaging heterozygous mutation at *DNMT3A* (p.R635W), was identified as an outlier sample in principle component (PC) 9 and 13. (**b**) Sample 2-0028-003, which carries a *de novo* frameshift mutation at *ADNP* (p.Q345fs), was identified as an outlier sample in PC9 and PC11 (and PC14 not shown). (**c**) Sample 2-1280-003 was identified as an outlier sample in PC 12. No *de novo* mutation in known epigene was found, but there is a maternal inherited rare damaging missense mutation at *KMT5C* (p.R205Q). (**d**) Functional enrichment of genes involved in the PC9 responsible for the sample outliers. Functions from negative loadings are in blue and that from positive loadings are in red.

After normalisation and the removal of problematic probe array data, we performed principal component (PC) analysis on the samples. We generated up to 20 PCs and used the Grubbs test for the detection of outliers (see Materials and methods). For each PC, we also adjusted for covariates such as gender, ethnicity, age, blood cell composition, batch effects and array chip orders.^32^ After correcting for covariates, we identified three significant outlier samples (from 185, 1.6%) from five different PCs (Supplementary Table 11): 2-0028-003 was identified from three PCs, 2-1276-003 was identified from two PCs and 2-1280-003 was identified from one PC (Figure 4). Interestingly, 2-0028-003 carries a *de novo* damaging missense mutation at *DNMT3A*, a gene involved in *de novo* DNA methylation,^33^ which is also a risk factor for ASD.^7,34^ The other outlier, 2-1276-003, carries a *de novo* frameshift deletion at *ADNP*, known to be involved in chromatin remodelling^35^ and ASD-risk.^36^ Both 2-0028-003 and 2-1276-003 were outliers in PC9, which captured genes enriched for function in neuron differentiation, cell morphogenesis and chromatin organisation (Figure 4d). The third outlier, 2-1280-003, did not carry a detectable predicted damaging DNM in a gene related to epigenetic regulation, but instead a maternally inherited mutation predicted to be damaging was detected in *KMT5C*, a gene function as a histone methyltransferase.^37^ It is not clear if this inherited mutation in *KMT5C* would lead to the aberrant DNA methylation profile, but the PC data may guide additional genetic or functional testing (see Supplementary Figure 14).

## Discussion

We have conducted a comprehensive analysis of the distribution of DNM across the entire genome in ASD cases and controls and discovered a cadre of new germline rare genetic variants of relevance to ASD. Lower-resolution microarray and targeted sequencing studies have implicated rare mutations in non-coding genes like *PTCHD1AS1,*^38,39^ 5′-UTR of *MBD5,*^40^ introns of *NRXN1*^41^ and more complex regulatory structural variants,^39,42^ but here our unbiased WGS assessment of germline mutations implicate numerous functional elements involved in regulating gene expression and chromatin organisation (Figure 3 and Figure 4). We also found that a proportion (1.1%) of DNMs previously thought to be germline in origin^7^ were in fact likely somatic events. So far, there are no genome-wide somatic mutation profiles in controls that we can compare our data against, but our findings of somatic rates in ASD are comparable with a study of intellectual disability.^43^ Moreover, using targeted genes, it has recently been shown that there is an excess rate of somatic mutation found in the coding regions of ASD probands compared with their unaffected siblings.^44^

Our most surprising observation was the clustering of germline DNMs arising on the maternal chromosome. We hypothesise that the generation of a *de novo* CNV might disturb DNA repair,^22^ and this entire process may be influenced in a sex-dependent manner both in gametogenesis^23^ and ultimately in post-natal phenotypic expression.^5^ While the overall contribution of this novel mechanism of mutation to ASD needs to be determined through much larger studies, we did find one ASD case (3-0438-000) having two *de novo* events impacting the coding region of the known ASD-risk gene, *SYNGAP1* (Supplementary Table 5). The finding of only 1 such example from 192 cases studied (0.5%), suggests that all other known genetic mechanisms involved in ASD are rare, but it could increase when the mutational impact on the non-coding genome is better understood. Our data also suggest, in the clinical genetics setting, that characterizing *de novo* CNVs alone may be insufficient when attempting to understand a full-genotype and -phenotype correlation, and sequencing near the breakpoints or better yet WGS may be required.^12,42,45^ Applying other improved WGS technologies, such as long-read and linked-read sequencing, for SV identification, implied that our previous knowledge of SVs was rather primitive. Our understanding of the genetics of ASD may further improve as these methods start to be widely used.^46–48^

Given the increasingly appreciated importance of chromatin remodelling function in the pathology of ASD,^5,6^ we established a general method to connect aberrant methylation profiles detected by microarrays to DNMs in WGS data (which can also act as a functional evaluation of the DNMs). Here, in 2 out of 185 (or 1.1%) cases, we found DNMs directly affecting the coding regions of known genes, *DNMT3A*^34^ and *ADNP,*^36^ which control the epigenetic cascade. This same approach should be equally amenable to implicate non-coding regulatory elements, and downstream target regions or genes for a particular epigene, as well as to confirm the damaging effects of mutations. Moreover, environmental influences on the epigenome in ASD biospecimens could be monitored using this strategy.

Our study provides a framework of how to use WGS in the study of ASD. The early data arising lends further support for a multifactorial threshold model underlying ASD^49–51^ with all types of variation (SNV/indel/CNV in coding and non-coding DNA, germline, somatic, epigenetic) involved. Here, we focused on studying the impact of DNMs as an entry point into WGS data, but similar studies of rare and common-inherited genetic variants,^52^ as well as non-genetic factors, will now need to be assessed in larger cohorts to quantitate relative risks for ASD.

## Materials and methods

### Samples for WGS

We selected 200 unrelated trio families from a cohort of Canadian ASD families, based on the fact that the index case (proband) was the only affected individual in the family at the time of proband’s diagnosis (simplex families). Diagnosis was based on using the ADI-R, ADOS plus clinical evaluation.^7^ We also considered the availability of genomic DNA from whole blood and completeness of phenotype information. We obtained informed consent from all participants, as approved by the Research Ethics Boards at The Hospital for Sick Children, McMaster University and Memorial Hospital. We genotyped all the samples using high-resolution microarray platforms for the detection of CNVs.

### WGS and variant detection

We sequenced trio families (two parents and one proband). We extracted genomic DNA from all samples and sequenced them with Illumina Hiseq 2000 technology. We ligated the purified DNA fragments with adaptor oligonucleotides to form pair-end DNA libraries with an insert size of 500 bp. Sequencing depth and coverage for each sample is summarised in Supplementary Table 1. We aligned the filtered reads to the reference genome (build GRCh37) with the Burrows–Wheeler Aligner as a sorted binary alignment map (BAM) format. We performed local realignment and quality recalibration with the Genome Analysis Toolkit for each genome. Details of the procedure can be found in Supplementary Information.

### *De novo* SNV and indel detection

We considered the variants in the proband to be a candidate *de novo* SNV if it was not present at the same position in both his/her parents. We used ForestDNM method to detect *de novo* SNV calls and filtering method for indels in all trios as previously described.^7^ We validated all the exonic and a subset of non-coding *de novo* SNVs and indels using Sanger sequencing. Details can be found in Supplementary Information.

### *De novo* CNV and detection

We used Segseq^53^ and ERDS^54^ to detect potential *de novo* CNVs. We also used Meerkat^55^ to detect potential *de novo* SVs. Details of the procedures can be found in Supplementary Information. We validated all the detected putative *de novo* CNV and SV by quantitative-PCR and/or Sanger sequencing.

### Functional annotation of DNMs

We annotated the variant call format (vcf) using a custom pipeline based on ANNOVAR (November 2014 version).^56^ We defined genes from RefSeq gene models (hg19 genome build; downloaded from UCSC 12 February 2013). We annotated the genomic conservation at the variant position using UCSC PhyloP and phastCons for placental mammals and 100 vertebrates.^57^

For the functional impact of genic variants, we used predictors including SIFT,^58^ PolyPhen2,^59^ Mutation Assessor,^60^ Mutation Taster^61^ and CADD.^62^ We also expanded the annotation of non-coding regulatory sequence through implementation of splicing exon inclusion/exclusion predictions.^28^

We created filtering tiers and annotated each variant based on conservation and predicted impact on coding and non-coding sequence. Damaging tier 1 is defined as having odds ratio >1.5. Damaging tier 2 is defined as variants having odds ratio >2.5 (except LOF and missense variants) (Supplementary Table 8).

Damaging tier 1 genic variants include: (1) all LOF (stop gain+core splice site) variants; (2) all the missense (including stoploss) variants; (3) splicing (both intronic and exonic, excluding stop gain) negative variants, as predicted by SPIDEX with dPSI<−3.5; (4) all 5′ UTR variants and (5) 3′ UTR variants with PhastCons>0.

Damaging tier 2 genic variants include: (1) all LOF (stop gain+core splice site) variants; (2) missense variants with at least 5 out of 7 predictive programmes meeting damaging criteria: mammalian PhyloP⩾2.30, vertebrate 100 PhyloP⩾4.0, SIFT<0.05, Polyphen2⩾0.90, Mutation Assessor⩾1.9, Mutation Taster⩾0.5, CADD phred⩾15; (3) splicing (both intronic and exonic, excluding stop gain) negative variants, as predicted by SPIDEX with dPSI<−5; (4) all 5′ UTR variants; (5) 3′ UTR variants with PhastCons>0 and mammalian PhyloP>=1.5.

For non-genic variants, we annotated the vcf by overlapping the DNase I hypersensitive sites and chromatin states extracted from FANTOM enhancers^63^ and enhancers in developing foetal brain,^64^ ENCODE^29^ and Epigenomic Roadmap.^30^ Details of tracks extracted can be found in Supplementary Table 12. For the functional impact of variants, we used predictors including CADD, DeepBind,^65^ FunSeq^66^ and conservation score (PhastCons and mammalian PhyloP).

We annotated each variant based on the overlap of chromatin states, conservation and predicted impact on the non-coding sequence. We assigned damaging tiers based on their high burden in ASD cohort (294 combinations; FDR<0.2). Damaging tier 1 is defined as having odds ratio >1.5. Damaging tier 2 is defined as variants having odds ratio >2 (Supplementary Table 9).

Damaging tier 1 non-genic variants include:

(1) DeepBind loss; PCons>0—DeepBind predicted loss of transcriptional binding factor (wild-type reference binding score>=99.9% percentile of genome background distribution and variant binding score<=99% percentile of genome background distribution, additionally requiring mammal, PhastCons>0).

Damaging tier 2 non-genic variants include:

(1) DeepBind loss; PCons>0 (DHS)—DeepBind predicted loss of transcriptional binding factor in DNase I hypersensitive regions (ENCODE).

### GC content correction

Coverage difference is known to affect the number of variants detected (sensitivity), in which we have adjusted it from our statistics (using total overall variants detected). We have also shown that there is no bias on the underlying sequence context. The non-linear regional variability is known to affect regions in extreme GC content and repetitive regions. Both our data and the control data have the repetitive regions assessed for *de novo* variant detection using machine learning. For GC content, we indeed found minor but statistically significant difference between ours and the control data. By comparing the GC content flanking the *de novo* SNVs (50, 200, 500 bp) between our cases and the controls, we found a minor but statistical significant difference in GC content (with 50 bp having the highest bias) (Supplementary Figure 10). Therefore, we used a logistic regression test and used the 50 bp flanking GC content as a covariate to correct for the confounding effect:

$$\begin{array}{c} F\mathrm{ull}\;\text{model}:\;y=\beta 1\times x_{1}+\beta 2\times x_{2}+c\\ \mathrm{compared}\;with\;\text{GC-only}\;\text{model}:\;y=\beta 1\times x_{1}+c \end{array}$$

where

*x*_1_, GC content.

*x*_2_, membership to variant set (based on impact prediction and region-of-interest).

*c*, intercept.

### Burden and pathway analysis of annotated DNMs

We performed logistic regression test to determine the significance of higher burden of DNMs in cases than controls.^67^ We used all DNMs in exonic coding, UTR and intronic regions as the universe for genic variant comparison. We used all DNMs, except exonic and predicted splicing mutations, as the universe for non-genic variant comparison. We only counted once if a variant appeared more than once in different annotated categories (to avoid double-count for variants with multiple annotations).

For curated ASD-related gene sets, we used a Fisher’s Exact Test on the contingency table defined by the case and control variants groups intersected by the damaging and not-damaging variant groups (self-contained). For Gene-Ontology Function gene sets, we used the same approach by including gene sets with gene number between 50 and 1,200. Gene sets with all counts equal to zero could not be tested and were removed We extracted the Gene-Ontology gene sets from the National Cancer Institute at the US National Institutes of Health (NCI-NIH). We computed the FDR using the Benjamini–Hochberg procedure.

### DNA methylation array

We bisulfite-converted the genomic DNA from 185 samples using the EpiTect PLUS Bisulfite Kit (Qiagen, Valencia, CA, USA) according to the manufacturer’s instructions. We eliminated probes: (1) on the sex chromosomes, (2) containing single nucleotide polymorphisms and (3) with detection *P* values>0.05 in any of the samples from the study. We performed background subtraction using the ‘noob’ method from Methylumi^68^ and normalisation by ‘SWAN’.^69^ Details of the procedures can be found in the Supplementary Information.

### Methylation outlier sample test

We performed PC analysis using the R stats package (https://www.R-project.org) function procomp (with scaling and centering). We generated 20 PCs from the DNA methylation *β* values, the same was repeated on a randomized matrix to determine 20 sets of eigenvalues. We analysed the top 14 PCs, which have eigenvalues higher the maximum of each randomized eigenvalue. The corrected and uncorrected PC values for the samples follow a normal distribution (Supplementary Figure 15). We then performed Grubbs test for outlier detection. For each PC, we adjusted for covariates including gender, ethnicity, age, blood cell composition, batch effects and array chip orders. We estimated the blood cell composition based on the *β* values using celltypes450 (version 0.1) from R package.^70^ We consider a sample being an outlier when it has a Grubbs test FDR<0.1 both before and after covariate correction.

### Availability of data and materials

The sequence data can be accessed through the MSSNG database on Google Genomics (for access see https://research.mss.ng).

## Figures and Tables

**Table 1:** Summary of *de novo* SNVs contribution^a^

	*ASD (*n*=192)*	*Control (*n*=258)*	*odds ratio (*P*)*
*Germline*			
All	9,774^b^	11,020	—
Coding	193 (1.98%)	136 (1.23%)	1.38 (5.0×10^−3^)
			
*Predicted damaging*			
All	244	141	1.84 (8.0×10^−9^)
Coding	151 (61.9%)	97 (68.8%)	1.53 (1.3×10^−3^)^c^
Genic non-coding	55 (22.5%)	23 (16.3%)	2.59 (7.0×10^−5^)^c^
Non-genic non-coding	38 (15.6%)	21 (14.9%)	2.14 (4.3×10^−3^)^d^

Abbreviation: SNV, single nucleotide variant.

a Comparison was based on a logistic regression model with GC content correction (see Materials and methods).

b Somatic mutations (*n*=613) were removed.

c All exonic and intronic variants as the universe.

d All non-exonic variants as the universe.
