# Supplementary material for: Genome-wide characteristics of de novo mutations in autism
Source: NPJ Genom Med. 2016 Aug 3;1:16027–. doi: 10.1038/npjgenmed.2016.27 (PMC4980121; doi:10.1038/npjgenmed.2016.27)

Supplementary Figure 1

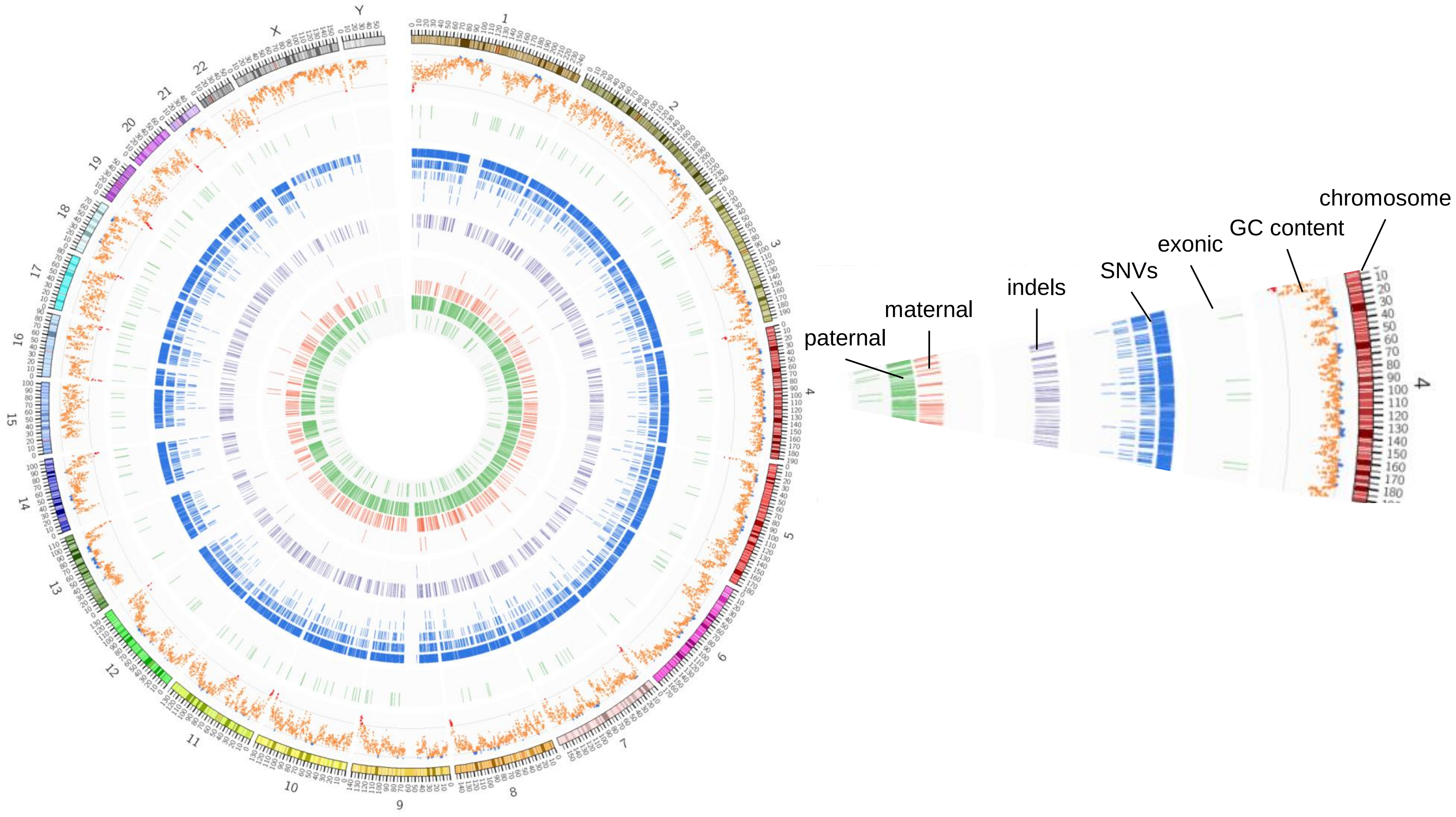

Supplementary Figure 2

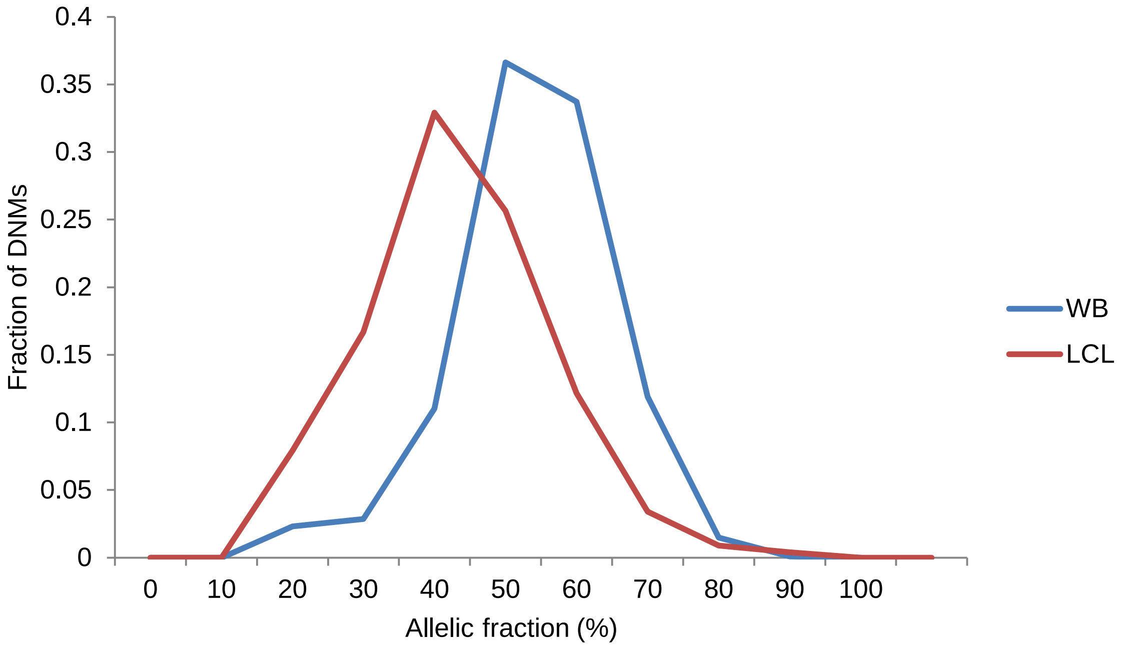

Supplementary Figure 3

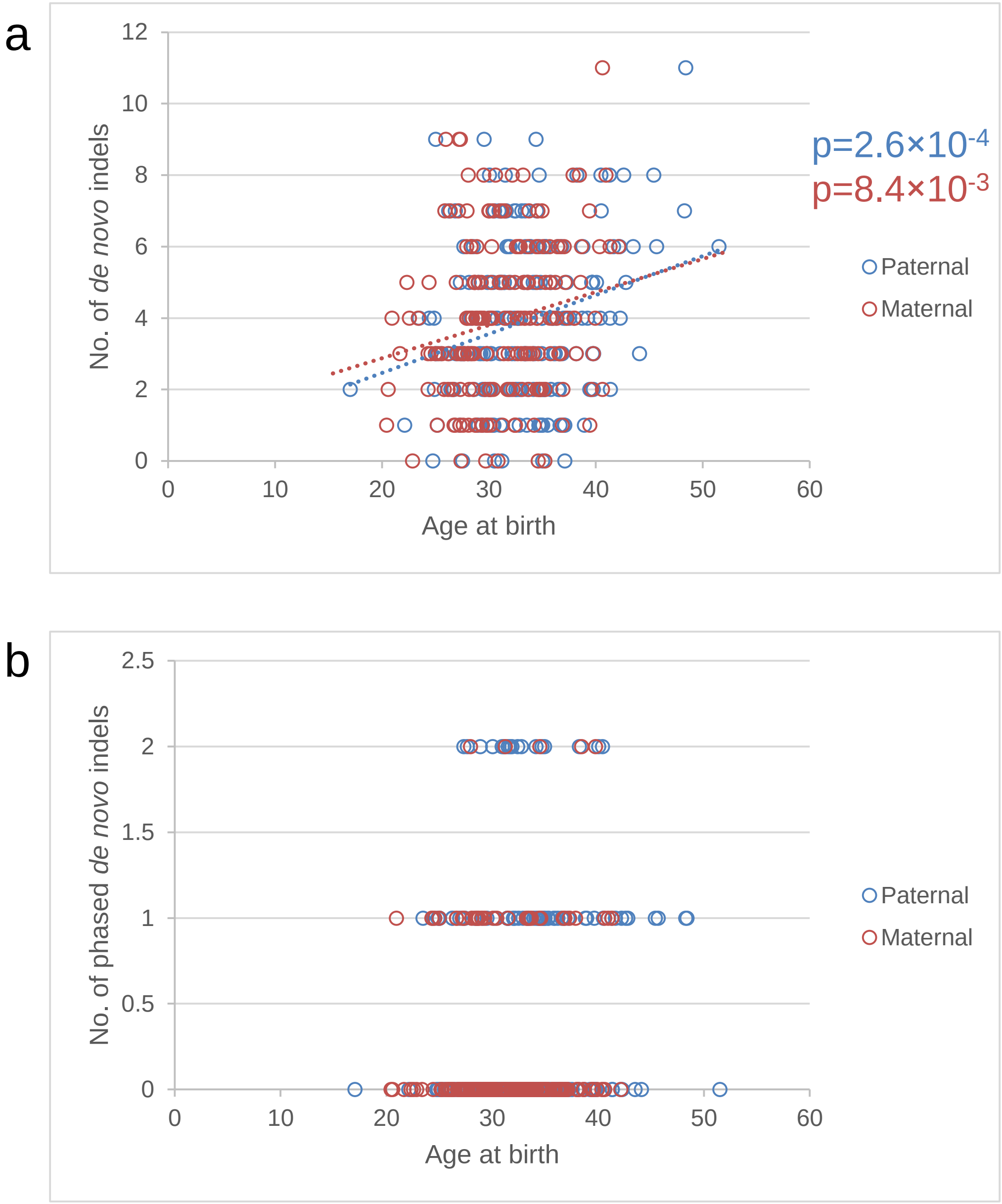

Supplementary Figure 4

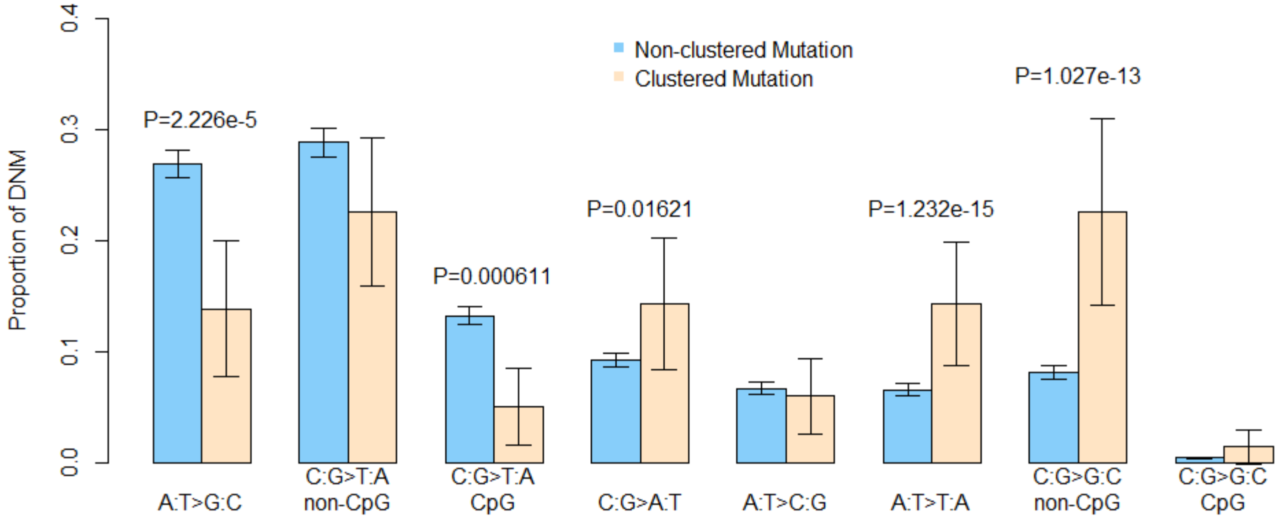

Supplementary Figure 5

a

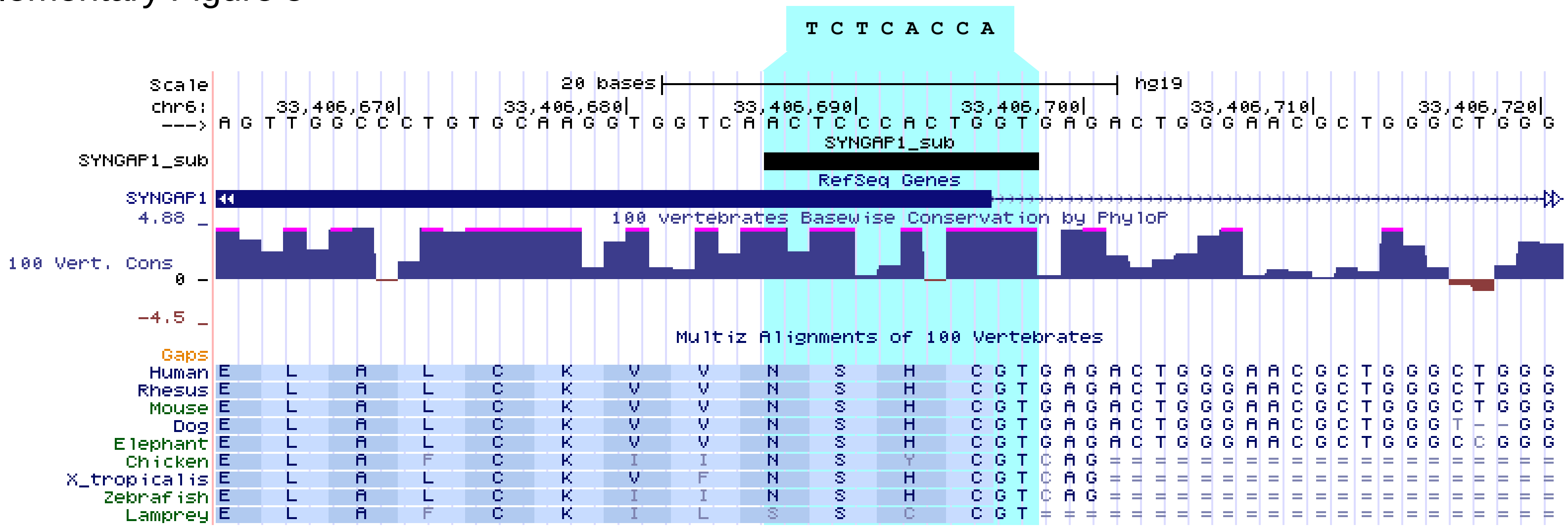

b

3-0438-100 mother

3-0438-101 father

3-0438-000 proband

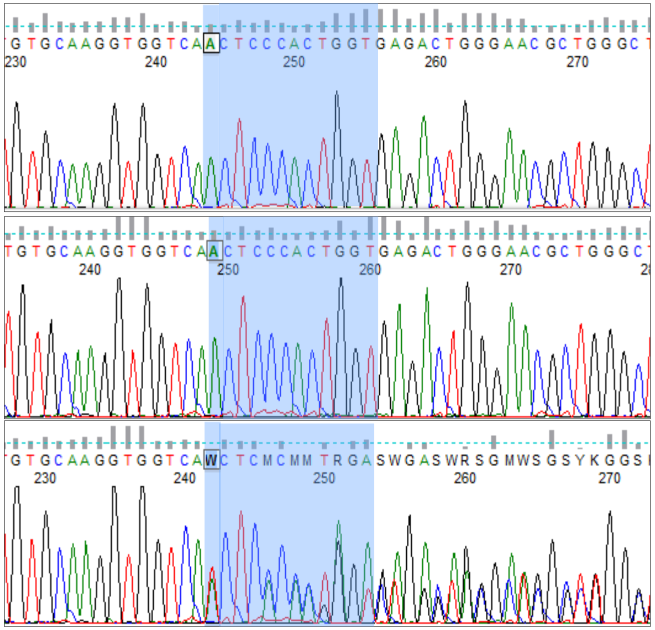

Supplementary Figure 6

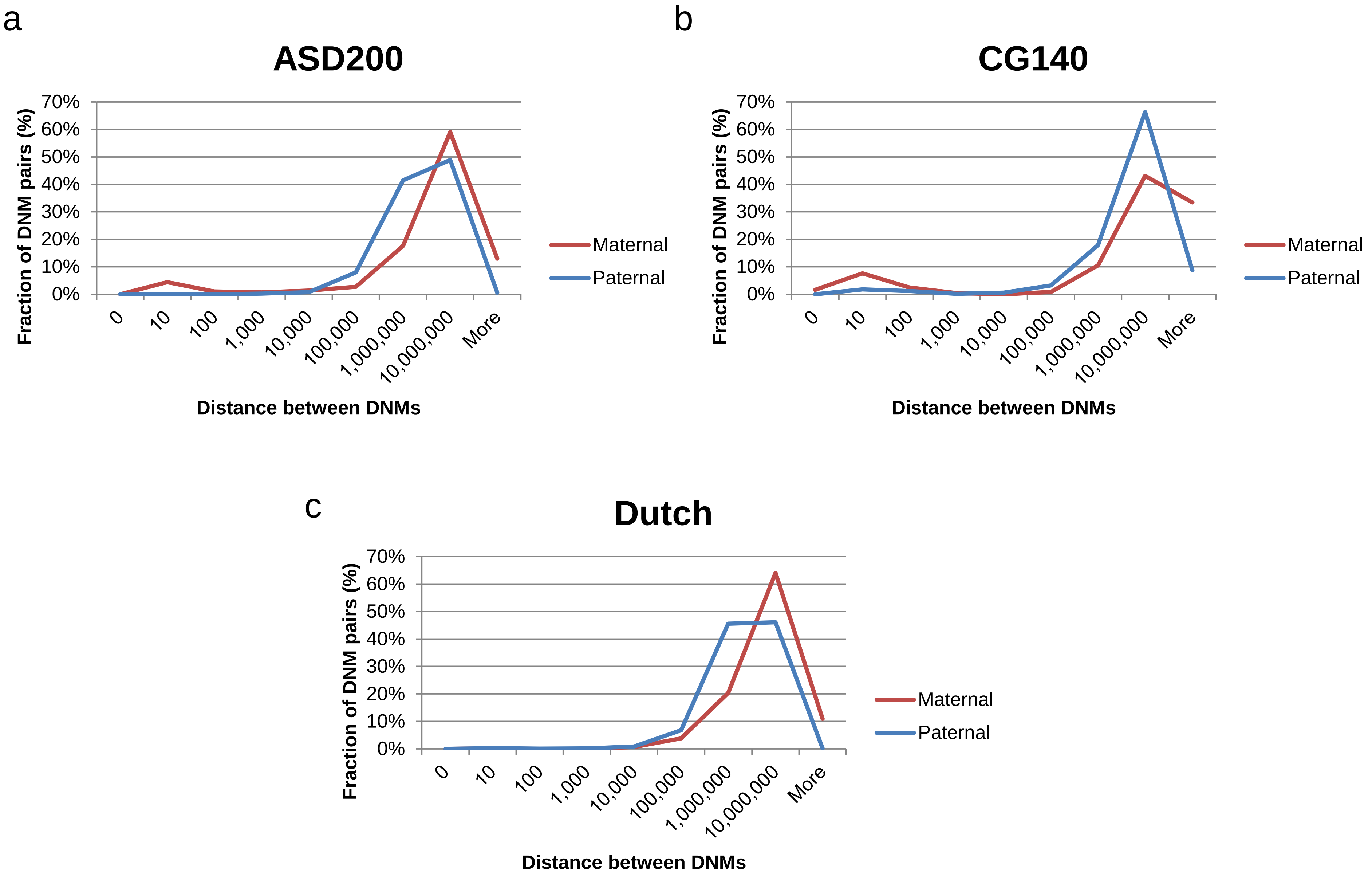

Supplementary Figure 7

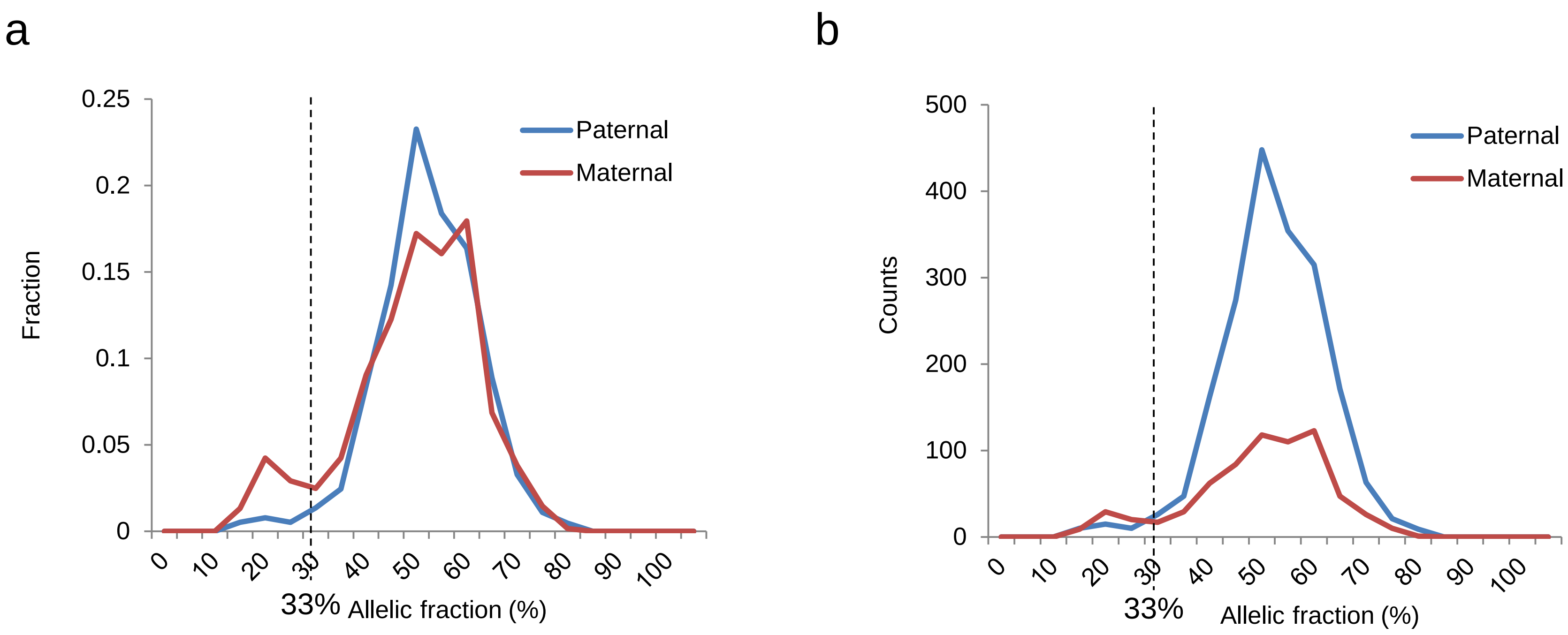

Supplementary Figure 8

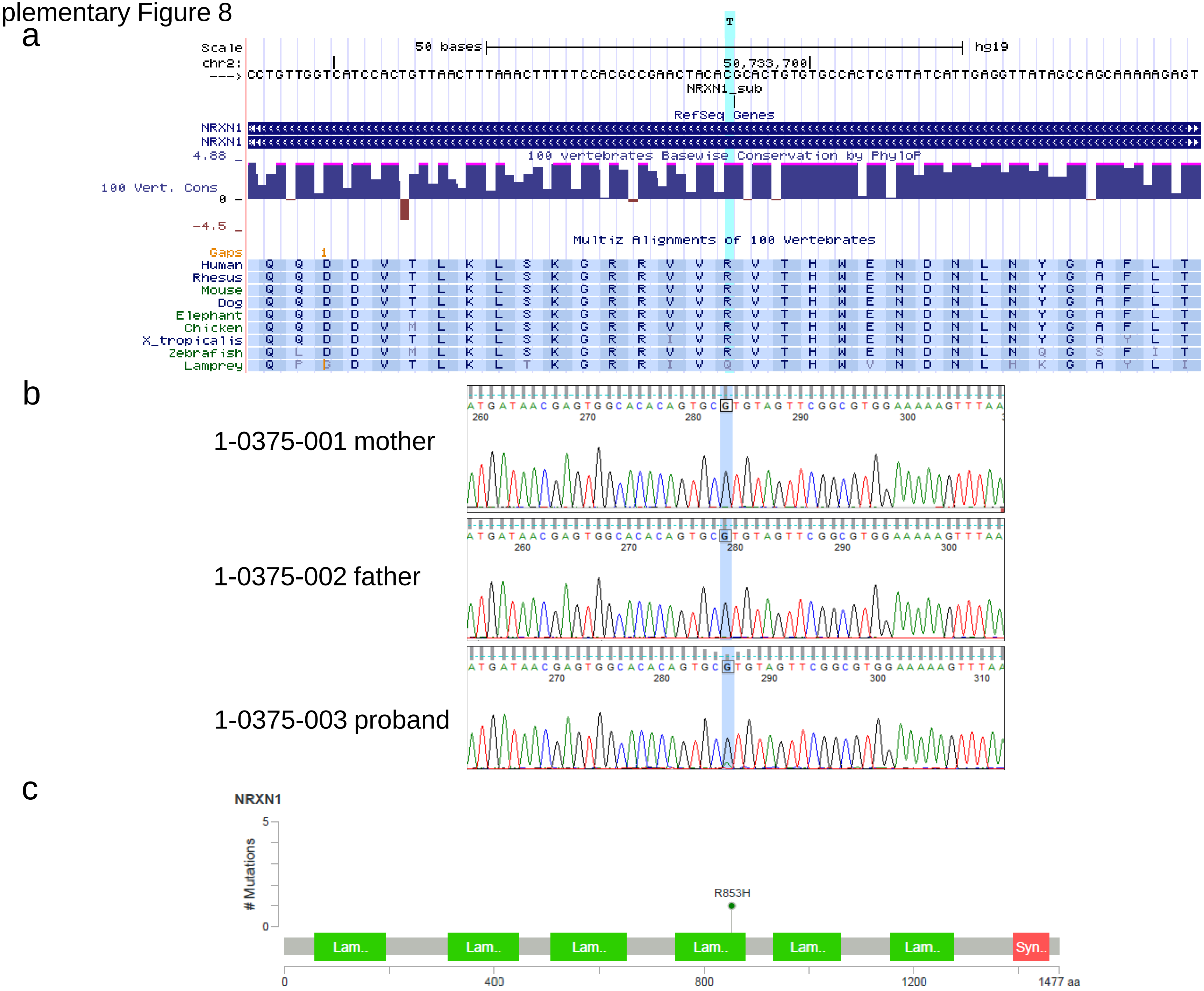

Supplementary Figure 9

a

	ASD somatic (25%)	ASD somatic (33%)	ASD	GoNL	Kong	CG	ASD LCL	CG LCL
ASD somatic (25%)	-	0.56	0.56	0.54	0.58	0.55	0.45	0.53
ASD somatic (33%)		-	0.64	0.61	0.64	0.40	0.40	0.60
ASD			-	0.94	0.84	0.83	0.36	0.62
GoNL				-	0.77	0.73	0.32	0.59
Kong					-	0.96	0.45	0.72
CG						-	0.40	0.67
ASD LCL							-	0.81
CG LCL								-

b

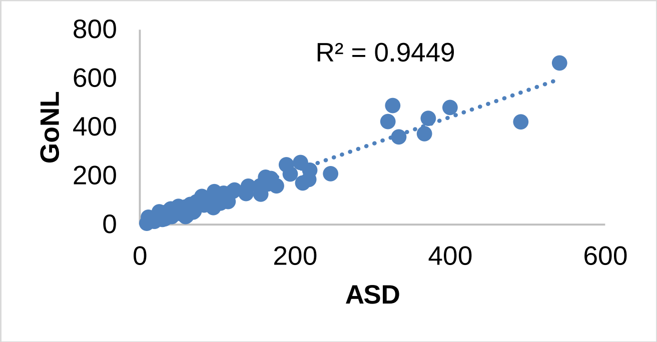

c

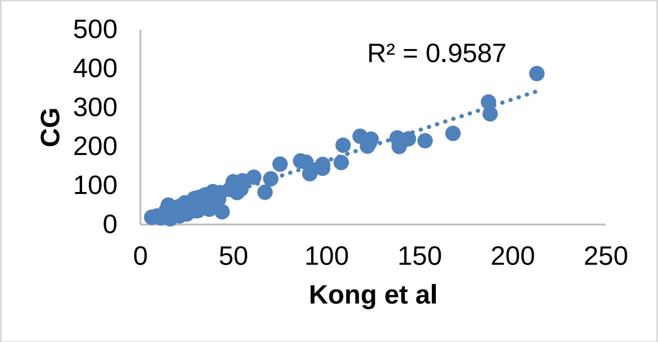

d

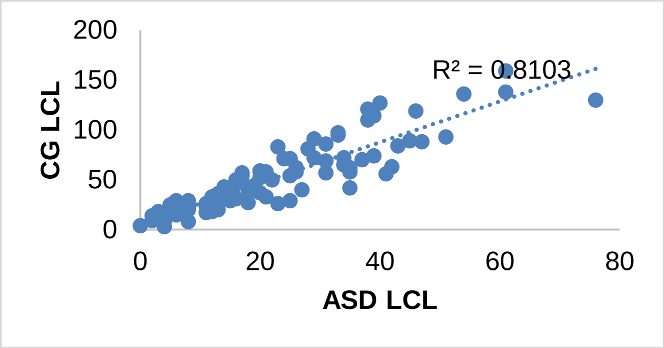

Supplementary Figure 10

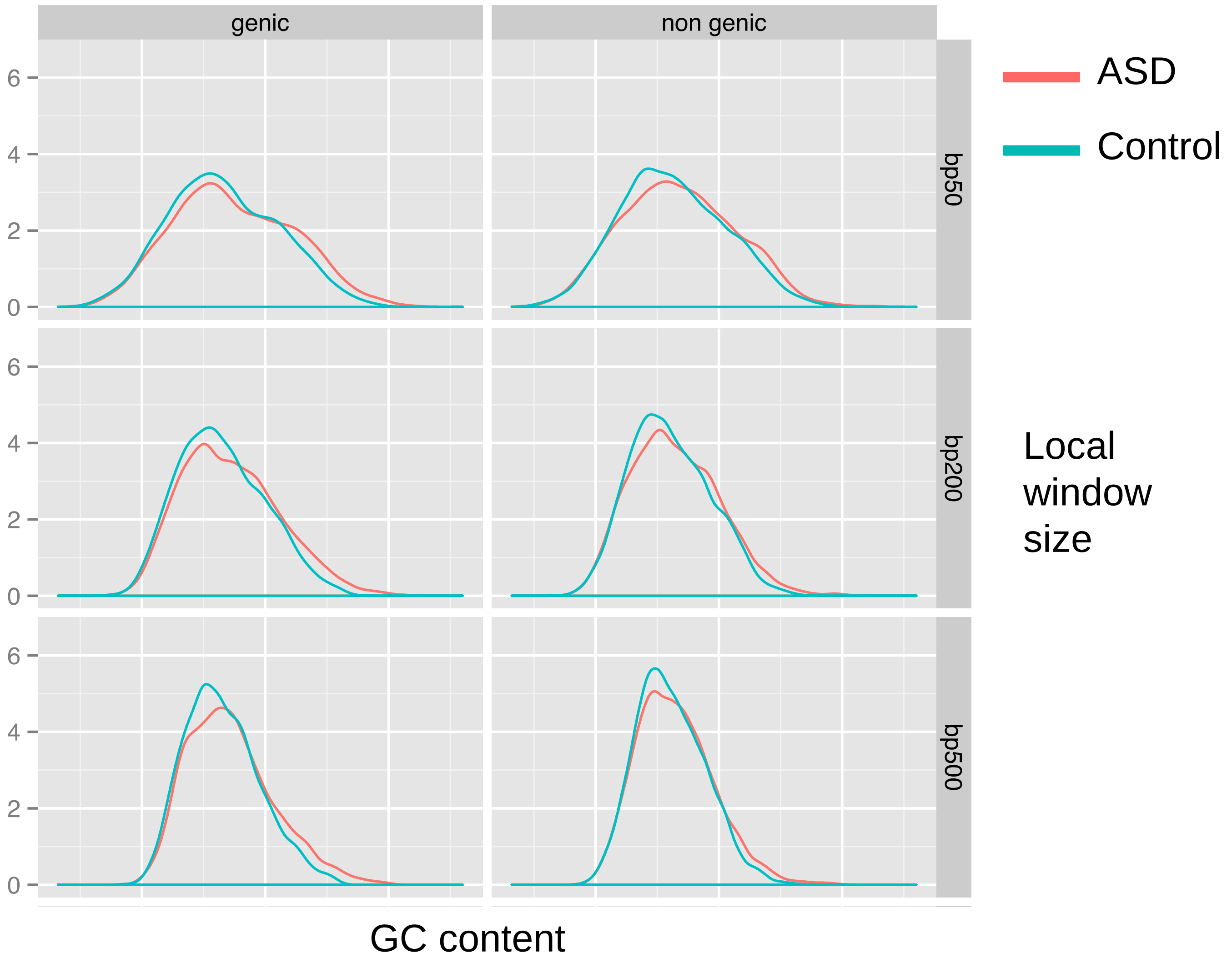

Supplementary Figure 11

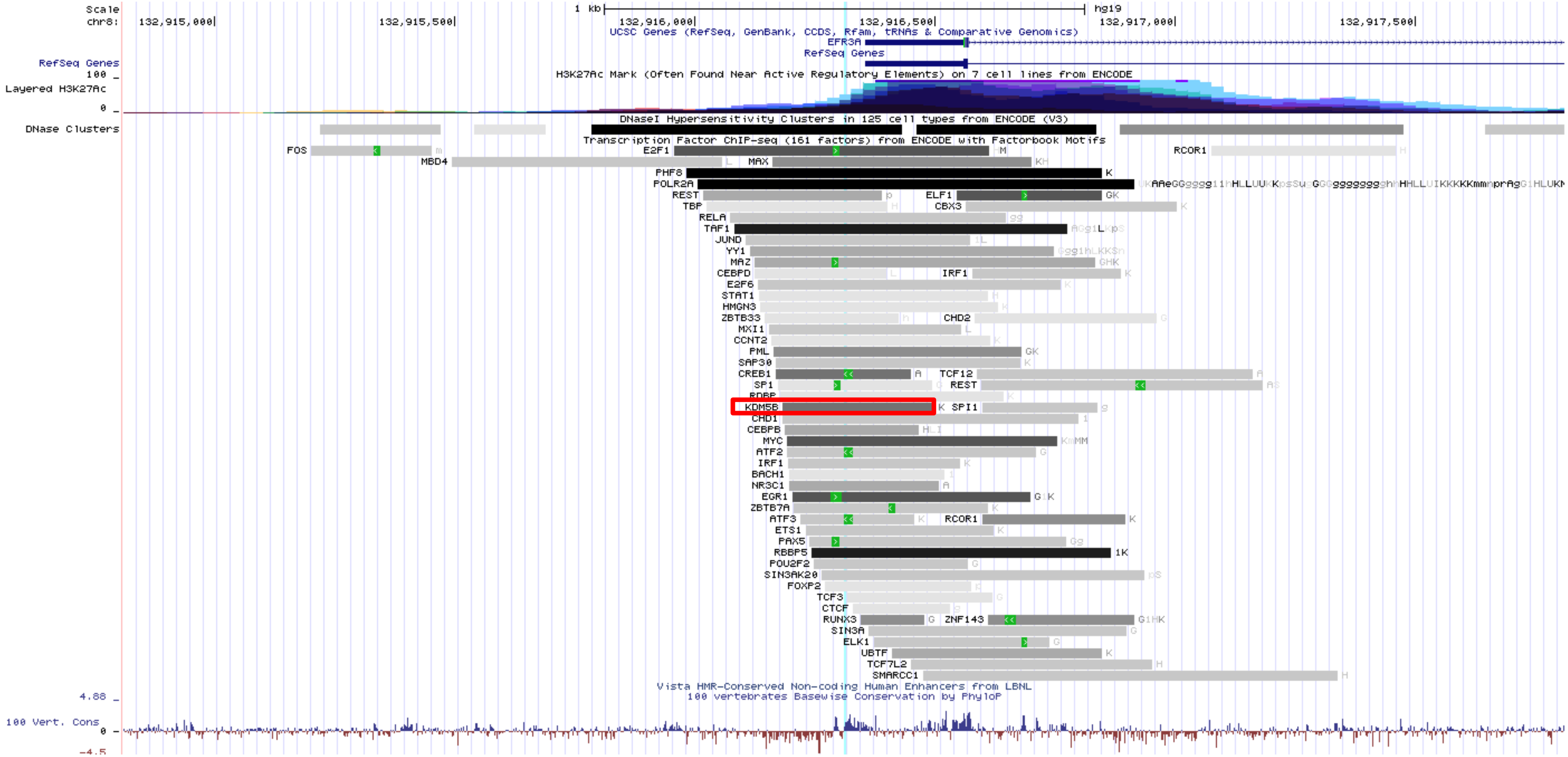

Supplementary Figure 12

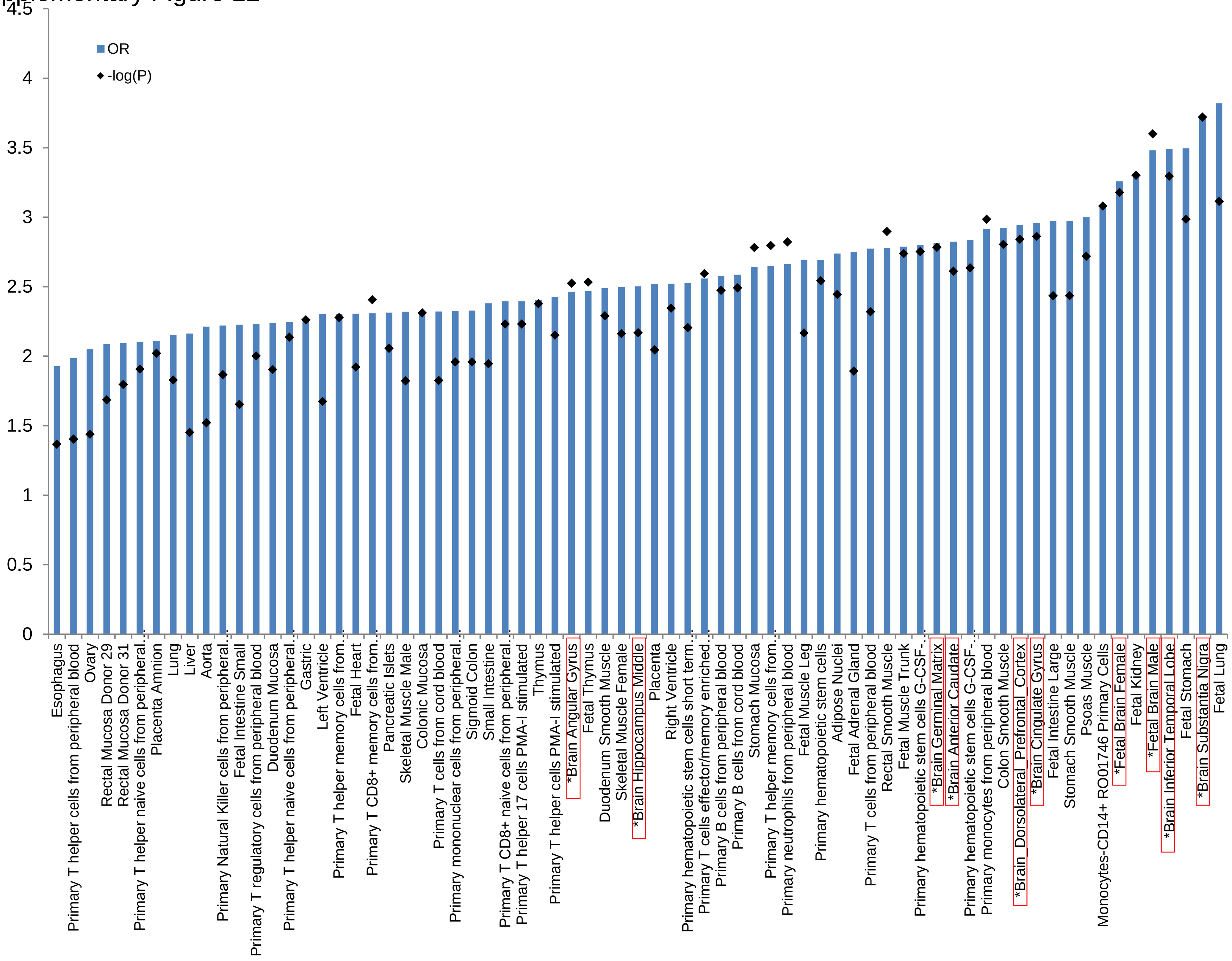

Supplementary Figure 13

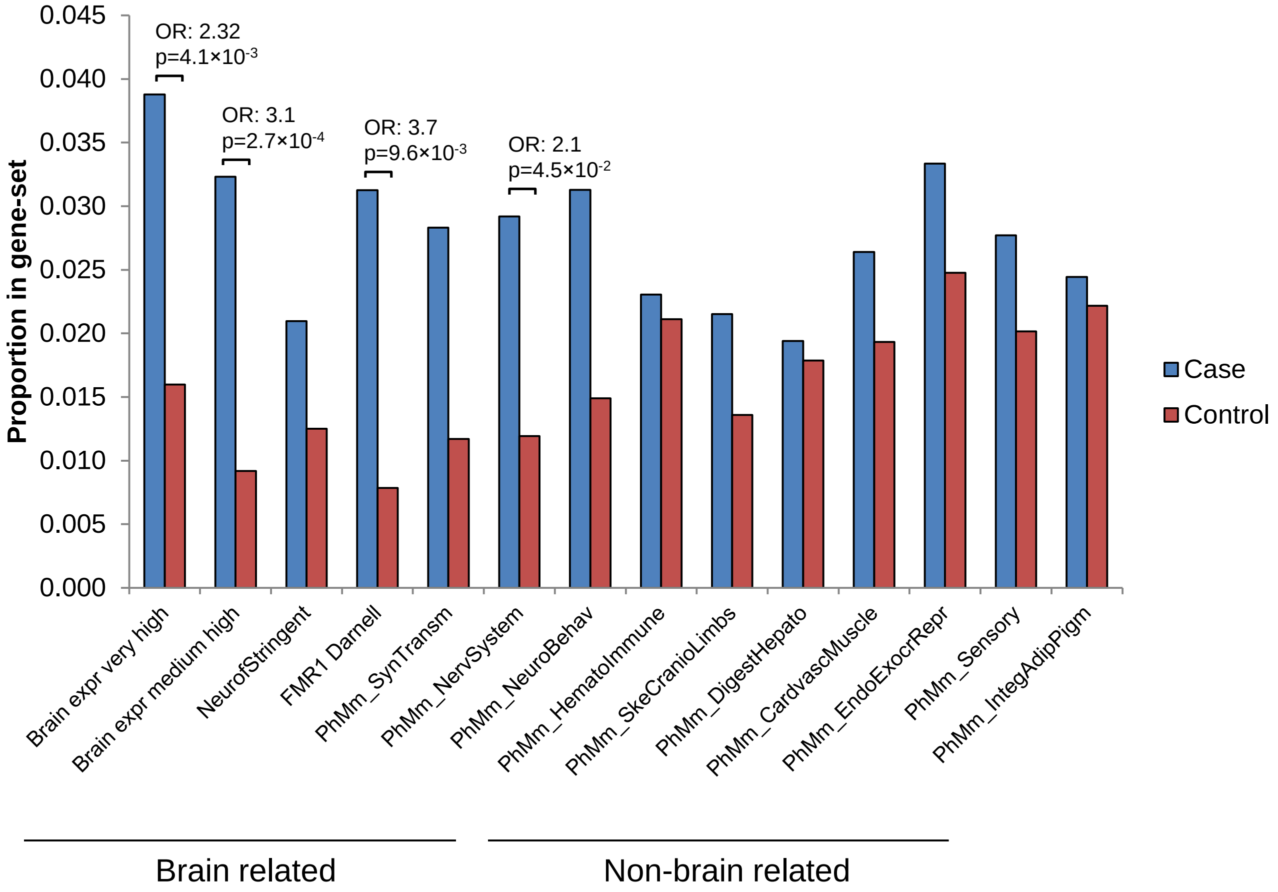

Supplementary Figure 14

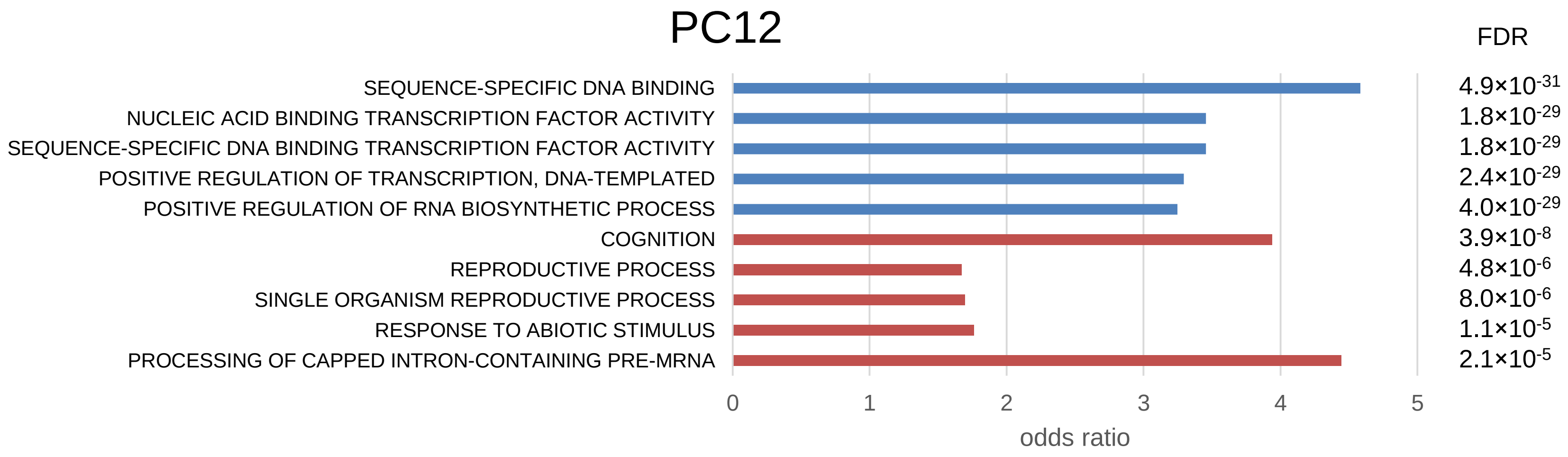

Supplementary Figure 15

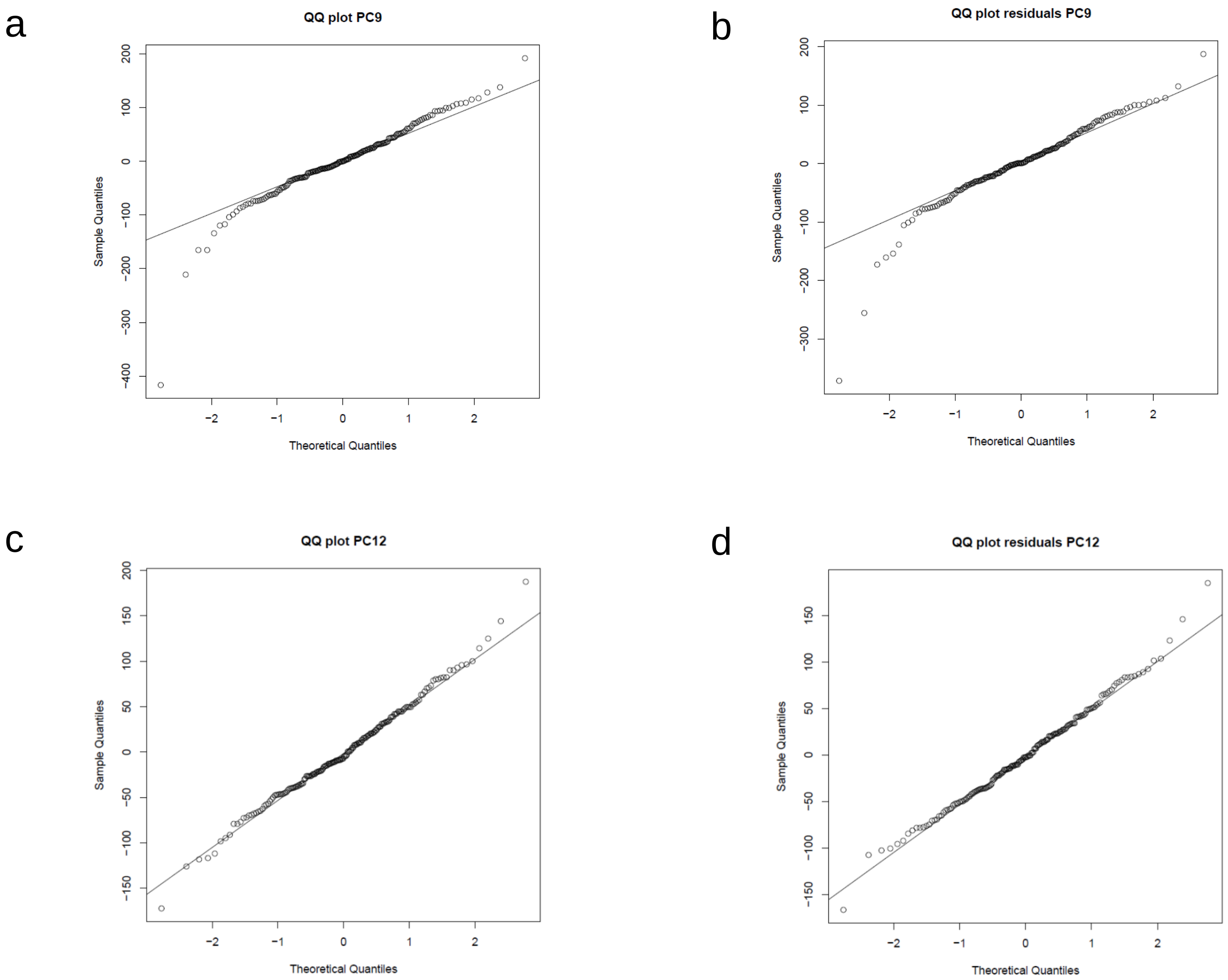

Supplement: Supplementary Information [file npjgenmed201627-s1.pdf]
