# Supplementary material for: Genome-wide characteristics of de novo mutations in autism
Source: NPJ Genom Med. 2016 Aug 3;1:16027–. doi: 10.1038/npjgenmed.2016.27 (PMC4980121; doi:10.1038/npjgenmed.2016.27)
Supplement: Supplementary Figures S1–S15 [file npjgenmed201627-s2.pdf]

Supplementary Notes

Variant detection

After whole genome sequencing, we removed the adaptor sequence and low quality bases from the sequence reads. We aligned the filtered reads to the reference genome (build GRCh37) with the Burrows-Wheeler Aligner (BWA v 5.9-r16 parameters: "aln -o 1 -e 63 -i 15 -IL -l 31 -k 2 -t 4 -q 10") as a sorted binary format (bam)[1]. We also realigned the reads in the major histocompatibility complex (MHC) region with eight different haplotypes from the reference genome. We selected reads that mapped to any of the MHC haplotypes and re-aligned them to the Chr6 PGF haplotype. We removed duplicated reads using Sequence Alignment/Map tools (SAMtools – v 0.1.18)[2]. We used only the uniquely mapped reads for variation detection. We performed local realignment and quality recalibration with the Genome Analysis Toolkit (GATK, version 1.6-13)[3] for each genome. We merged the bam files from the same trio family and detected variants using both Mpileup (SAMtools) and GATK. Consistent variant calls detected by both methods were considered to be confident calls. We performed local realignment and quality recalibration at the confident calling regions.

We detected single nucleotide variants (SNVs) and small insertions/deletions (Indels) using GATK (UnifiedGenotyper) [3 33] for all trios jointly, i.e., the realigned reads of the three members in a trio family were used simultaneously as inputs. We detected CNVs using "estimation by read depth with single-nucleotide variants" (ERDS) (version 1.1 default parameters)[4] and Segseq (v 1.0.1)[5] for each genome. We also detected structural variation (SV) using Meerkat (version 0.184)[6]. We annotated the effects (e.g., missense, nonsense, or frameshift mutations) and classifications (e.g., in exonic, intronic, or intergenic regions) of variants across the genome using ANNOVAR (version 20130211)[7]. The output file was generated in the universal Variant Call Format (vcf). Numbers of SNVs and indels detected per sample are summarized in Supplementary Table 1.

De novo SNV detection

We considered a variant in the proband to be a candidate *de novo* SNV if it was not present at the same position in either parent. We generated an initial list of variants that were inconsistent with Mendelian inheritance using GATK. We used the ForestDNM method to remove false positive calls and refine the candidate *de novo* SNV calls in all trios[8]. We used an additional filtering method to detect potential *de novo* SNV with the following criteria: a) The call in the proband was removed if more than 70% of reads were called as heterozygous reference, or if more than 5% of non-reference reads occurred in either parent (indicates that the parent is likely a mis-called homozygous reference); b) The call in the proband was considered spurious and removed if its sequencing depth was less than 10% of the total sequencing depth of his/her parents at the corresponding site; c) All the sites that were

located within the Indel regions +/- 5 bp were excluded; d) The 'phred-scale' filter (PL ≥30 for the genotypes of the proband and both parents) based on likelihood of the genotype was further applied to refine the candidates; e) If the HomopolymerRun (Hrun: largest contiguous homopolymer run of the variant allele in either direction on the genome reference) of the site is less than 7, it was included.

De Novo Indel Detection

We applied similar filtering criteria for *de novo* indel detection: the proband had to be heterozygous for the indel call, whereas the parents both had to be homozygous reference at the same position. The read depth at the variant position of each family member was more than 10×. Furthermore, the variant calls in the trio data were selected with the following filters: a phred-scaled quality score (QUAL) of more than 30; a QualByDepth (QD: variant confidence from the QUAL field/unfiltered depth) of more than 10; a HomopolymerRun less than 5; and a MappingQualityZero (MQ0: total count across all samples that had reads with a mapping quality of zero) less than 4. In addition to the essential variant-quality filters, the indel call from the proband needed to be supported by at least 30% of the reads, and it needed to be with at least 15X read depth. Also, parents could have no reads with an indel at the same position where an indel was detected in the proband. Since *de novo* indels found in database SNP (dbSNP) and simple repeats were prone to be false positives, we filtered out indels reported in dbSNP137 and those located in simple repeat regions.

Additional filtering for *de novo* SNVs and Indels

We developed an additional filtering strategy to refine the detection of *de novo* SNVs and Indels by checking how often the sites were called in the parents (400 individuals as internal allele frequency). We extracted the SNV/Indel calls in the region corresponding to +/- 5 bp of each putative *de novo* call from each parent's vcf file. We then computed the alternate allele frequency of each given region in the parents, using vcftools. For putative *de novo* SNVs, we applied allele frequency = 0 for each locus where a dbSNP was present (tended to be a common SNP), and ≤1 where no dbSNP was found. For putative *de novo* indels, we applied allele frequency = 0. In addition, we filtered out variants with more than three putative *de novo* SNVs/Indels in the same haplotype.

Validation of *de novo* SNVs and indels

We used Primer 3 to design primers to span at least 100 bp upstream and downstream of a putative variant. In designing primers, we avoided regions of repetitive elements, segmental duplication or known SNPs. We randomly selected putative *de novo* SNVs from the whole genome sequencing data of two probands (2-1266-003 (50 variants) and 3-0141-000 (77 variants)) in the trio families (Supplementary Table 2). By Sanger sequencing we validated all the exonic *de novo* SNVs and indels from all trios (except 4 variants for which we were unable to design primers), using DNA from whole blood.

Candidate regions were amplified by PCR for all trios and assayed by Sanger sequencing.

Phasing *de novo* SNVs and indels

In order to identify the parent-of-origin for the *de novo* SNVs, we first assigned the parental origin of all the inherited SNVs using BEAGLE[9]. We extracted the inherited SNVs from the vcf. We used only the SNVs from a trio in which the quality filter was marked as "PASS", "TruthSensitivityTranche99.00to99.90" or "TruthSensitivityTranche99.90to100.00". We then used the ReadBackedPhasing algorithm from GATK for local phasing of the *de novo* SNVs, based on vcf and bam files. For *de novo* indels, we used DenovoGear to determine the parent-of-origin. We assessed the quality of phasing by manually checking the evidence of the reads from bam files supporting the phase results.

De novo CNV detection

To detect potential *de novo* CNVs, we used Segseq (for detection of large CNVs with more than 10kb in size), and ERDS (for detection of small CNVs with less than 10kb in size).

Since Segseq requires control references, we used the father (F) and mother (M) of the proband (P) for comparison (namely P-F, P-M) within each trio. We considered the CNV calls present in the proband only, in both P-F and P-M. From these, we selected the candidate *de novo* CNVs according to the following criteria: the CNV detected in the proband from P-F and P-M needed to 1) have at least 50% reciprocal overlap; 2) be the same CNV type (i.e., duplication or deletion); 3) have less than 1.5-fold of copy number difference.

To further reduce the false detection of *de novo* CNVs, we removed those present in Database of Genomic Variants (DGV). We also discarded CNVs that were present more than twice in the 200 probands. We retrieved the read depth distribution and manually inspected each candidate, considering it as a *de novo* candidate when it passed all above criteria.

For the ERDS method, we detected CNVs in individual samples using default parameters. We removed all the CNVs containing N-regions and obtained CNV sets for each individual from each trio. We identified putative *de novo* CNVs that were present in the proband but not in either parent. We then filtered out CNVs that were present in DGV. We filtered out CNVs that were present more than twice among the 200 probands. We also manually inspected the read depth-distribution to refine the list of *de novo* CNVs detected.

De novo SV detection

For *de novo* SV detection, we applied Meerkat (somatic calling function) for each proband, using either parent as control sample, with parameters recommended in the manual (-n 1 -D 5 -u 1 -f 1 -e 1 -z 1 -d 40 -t 20), to identify two 'somatic SV events' sets named P-F and P-M. We then intersected P-F and P-M to identify initial candidate *de novo* SVs. As an alternative approach, we used Meerkat to individually call SVs for each sample in each trio (parameters set as -d 5 -c 5 -p 3 -o 1), and then identified candidate *de novo* SVs that were present only in the proband. For all the candidate *de novo* SVs, we retained those that: 1) were larger than 100bp and smaller than 1Mb, 2) were supported by at least 2 read clusters, 3) had both breakpoints not from satellite or simple repeats, 4) had less than 40bp homology or unmatched nucleotides at either breakpoint.

We further removed SVs that were present in DGV (not only position-based but also SV type-based) and those that recurred among the 200 probands. We checked the depth distribution of SVs, and used matchclips[10] to double validate if there were clipped reads supporting the SVs. SVs without evidence from depth distribution and matchclips were eliminated. For all types of the candidate *de novo* SVs, we manually inspected the discordant reads distribution through Integrative Genomics Viewer (IGV).

De novo CNV validation

We confirmed all the detected putative *de novo* CNV by quantitative-PCR (qPCR) and/or Sanger sequencing. For qPCR, we designed two independent assays encompassing two regions in each candidate CNV. We tested all DNA samples from the trio family using Sybr-Green (Stratagene). We performed each assay in triplicate for both the target region probe-sets and the control region probe-sets. We determined the relative dosage ratio between target region and control region by comparing the cycle threshold and standard curve.

Phasing *de novo* CNVs and SVs

Parental origin was determined for each *de novo* CNV/SV by analyzing the transmission of SNPs within the region from each parent to the child. For duplications, we selected heterozygous variants with B-allele frequency (BAF) greater than 0.6 or less than 0.4 in CNV regions, and determined the duplicated allele. For all *de novo* CNVs in which informative SNPs existed, allele inheritance was consistent with inheritance from one parent only. For deletions, SNPs from the remaining allele were used for phasing. We also predicted the mechanisms of formation by BreakSeq[11].

DNA methylation array

We bisulfite-converted the genomic DNA from 185 samples using the EpiTect PLUS Bisulfite Kit (Qiagen, CA, USA) according to the manufacturer's instructions.

Bisulfite treatment converted unmethylated cytosines to uracils while leaving methylated cytosines intact. We randomly arrayed the bisulfite-converted DNA samples and subjected them to the Infinium HumanMethylation450 BeadChip panel array-based assay (Illumina, San Diego, CA). The array interrogates 482,421 CpG sites (21,231 RefSeq genes) in the human genome. The methylation level for each CpG site was measured by the intensity of fluorescent signals corresponding to the methylated allele (Cy5) and the unmethylated allele (Cy3). Continuous β values, from 0 (unmethylated) to 1 (methylated), were used to identify the percentage of methylation for each CpG site. The β value was calculated based on the ratio of methylated/(methylated + unmethylated) signal output. We eliminated probes: 1) on the sex chromosomes, 2) containing SNPs, and 3) with detection P values >0.05 in any of the samples from the study.

Supplementary Figure Legends

Extended Data Figure 1. Circos plot of genomic distribution of germline de novo mutations from ASD individuals. "paternal": paternally derived de novo mutation, "maternal": maternally derived de novo mutation, "indels": de novo indels, "SNVs": de novo single nucleotide variants, "exonic": exonic de novo mutations.

Supplementary Figure 2. Distribution of *de novo* mutations with respect to allelic ratio for the *de novo* mutations from different DNA sources. Allelic fraction is defined as the number of reads supporting an alternative allele to the number of common allele reads at a particular locus.

Supplementary Figure 3. Distribution of maternal and paternal *de novo* mutations with respect to allelic fraction. (a) Proportion of DNMs in different allelic ratio. (b) Counts of DNM in different allelic fraction. Dashed lines indicate the cutoff of to define somatic mutations (less than 33% allelic fraction).

Supplementary Figure 4. Location and validation of a somatic mutation at *NRXN1*. (a) Genomic location of the variant. A cytosine to tyrosine missense substitution was found at a conserved position in *NRXN1*. (b) Sanger sequencing of mother, father and proband confirmed a somatic mutation in the proband (1-0375-003). (c) Location of the somatic mutation in the NRXN1 protein. The mutation leads to an amino acid change from arginine to histidine at position 853 (NM_001135659).

Supplementary Figure 5. Correlation of *de novo* indels with age of parents. (a) Correlation of total number of *de novo* indels with the age of parents at birth of the child. (b) Correlation of number of phased *de novo* indels with the age of parents at birth of the child.

Supplementary Figure 6. Proportion of clustered and non-clustered *de novo* mutations in various classifications of nucleotide change.

Supplementary Figure 7. Location and validation of a *de novo* mutation cluster at *SYNGAP1*. (a) Genomic location of a 12bp to 7bp substitution at a conserved position in *SYNGAP1*. (b) Sanger sequencing of mother, father and proband confirmed the substitution in the proband (3-0438-000).

Supplementary Figure 8. Distance between *de novo* mutations according to parent of origin. (a) Proportion of *de novo* mutations (DNMs) in the present ASD cohort with certain distance measured between two DNMs. (b) Proportion of *de novo* mutations (DNMs) in the previous ASD cohort with certain distance measured between two DNMs. (c) Proportion of *de novo* mutations (DNMs) in the Dutch population cohort with certain distance measured between two DNMs.

Supplementary Figure 9. Correlation of sequence context between studies and variant types. (a) Correlation coefficients between different studies and variant types (somatic or germline). “ASD somatic (25%)”; somatic mutations defined by < 25% allelic ratio; “ASD somatic (33%)”: somatic mutations defined by < 33% allelic ratio. Variants in “ASD somatic (25%)” category have a higher correlation coefficient with ASD lymphoblast derived cell line (LCL) than variants in “ASD somatic (25%)” do, presumably due to higher proportion of somatic mutations in “ASD somatic (25%)”. “ASD”: germline de novo mutations from the ASD samples in our present study; “GoNL”: de novo mutations reported in the Dutch Genome of the Netherlands (controls); “Kong”: DNMs reported in Kong et al. Nature 2012; “CG”: DNMs we identified in our previous ASD cohort (Yuen et al 2015 Nature Medicine); “ASD LCL”: DNMs in the lymphoblast-derived cell-line (LCL) in the present ASD cohort; “CG LCL”: DNMs in the LCL in our previous ASD cohort (Yuen et al 2015 Nature Medicine. (b) Correlation of sequence context between DNMs detected in the present ASD cohort and the Dutch control cohort. (c) Correlation of sequence context between DNMs in Kong et al (2012 Nature) and our previous ASD cohort (Yuen et al 2015 Nature Medicine). (d) Correlation of sequence context between the DNMs detected in LCL in the present ASD cohort and our previous ASD cohort (Yuen et al 2015 Nature Medicine).

Supplementary Figure 10. GC content at flanking regions of DNMs. Distribution of GC content at the position of DNMs with different size of flanking sequence (50bp, 200bp and 500bp) is shown for genic and non-genic regions.

Supplementary Figure 11. Predicted loss of transcriptional factor binding in the promoter region of *EFR3A*. UCSC Genome Browser view of the location where DeepBind predicted a

loss of binding of transcriptional factor, KDM5B. The region overlaps H3K27Ac Mark and DNaseI hypersensitivity sites.

Supplementary Figure 12. Enrichment of DNMs predicted with loss of transcriptional factor binding in brain regions. From left to right: ascending order of odds ratios for predicted loss of transcriptional factor binding effect in in quiescent states of 71 different cell types or tissues. Brain-related cell types or tissues were indicated with red boxes.

Supplementary Figure 13. Burden of *de novo* mutations in ASD cases and Dutch controls. “Brain expr very high”: genes with at least 5 BrainSpan data points for which log2 (rpkm) >= 4.86. “Brain expr medium high”: genes with at least 5 BrainSpan data points for which 4.86 > log2 (rpkm) >= 3.32. “NeurofStringent”: genes in at least two of the curated Gene Ontology and pathway derived sets of neurobiological relevance. “FMR1Darnell”: human orthologs (NCBI Homologene) of mouse genes whose mRNA translation in neurons is likely to be regulated by the FMR1 protein, based on crosslinking immunoprecipitation (HITS-CLIP) of mouse brain polyribosomal mRNAs. PhMm: genes whose knock-out (or other genetic construct) produces a phenotype in mouse, downloaded from MGI (Mouse Genome Informatics); SynTransm: Synaptic Transmission; NervSystem: Nervous System; NeuroBehav: Neurobehaviour; HematoImmune: Hemato-immune; SkeCranioLimbs: Skeletal-limbs; DigestHepato: Digestive-hepatic; CardvascMuscle: Cardio-muscle; EndoExocrRepr: Endo-exocrin; PhMm_Sensory: Sensory; PhMm_IntegAdipPigm: Adipo-integument.

Supplementary Figure 14. Functional enrichment of genes involved in the Principle Component (PC) 12 responsible for the sample outliers. Functions from negative loadings are in blue and that from positive loadings are in red. The extreme 1% of probes with highest (for positive) or lowest (for negative) PC loadings were compared against randomly picked probes from the PC loading ranked between 40% and 60% (with total number adjusted to match the total number from the top 1% probes). Numbers of genes involved in the two sets of probes were compared using Fisher’s Exact test. P value was adjusted using Benjamini Hochberg false discovery rate (FDR) method.

Supplementary Figure 15. QQ plots of uncorrected and corrected values for samples in Principle Component 9 and 13. Distribution of eigenvalues of samples for both PC9 and 12 before (a and c) and after confounders correction (b and d). Values of samples in all PCs were under normal distribution after removing detected outliers (based on normality test).

Reference

1. Li, H. & Durbin, R. Fast and accurate short read alignment with Burrows-Wheeler transform. *Bioinformatics* **25**, 1754-1760 (2009).
2. Li, H., *et al.* The Sequence Alignment/Map format and SAMtools. *Bioinformatics* **25**, 2078-2079 (2009).
3. McKenna, A., *et al.* The Genome Analysis Toolkit: a MapReduce framework for analyzing next-generation DNA sequencing data. *Genome Res* **20**, 1297-1303 (2010).
4. Zhu, M., *et al.* Using ERDS to infer copy-number variants in high-coverage genomes. *American journal of human genetics* **91**, 408-421 (2012).
5. Chiang, D.Y., *et al.* High-resolution mapping of copy-number alterations with massively parallel sequencing. *Nat Methods* **6**, 99-103 (2009).
6. Yang, L., *et al.* Diverse mechanisms of somatic structural variations in human cancer genomes. *Cell* **153**, 919-929 (2013).
7. Wang, K., Li, M. & Hakonarson, H. ANNOVAR: functional annotation of genetic variants from high-throughput sequencing data. *Nucleic Acids Res* **38**, e164 (2010).
8. Jiang, Y.H., *et al.* Detection of Clinically Relevant Genetic Variants in Autism Spectrum Disorder by Whole-Genome Sequencing. *American journal of human genetics* (2013).
9. Browning, B.L. & Browning, S.R. A fast, powerful method for detecting identity by descent. *American journal of human genetics* **88**, 173-182 (2011).
10. Wu, Y., Tian, L., Pirastu, M., Stambolian, D. & Li, H. MATCHCLIP: locate precise breakpoints for copy number variation using CIGAR string by matching soft clipped reads. *Front Genet* **4**, 157 (2013).
11. Lam, H.Y., *et al.* Nucleotide-resolution analysis of structural variants using BreakSeq and a breakpoint library. *Nat Biotechnol* **28**, 47-55 (2010).
